# Pristimerin Suppresses RANKL-Induced Osteoclastogenesis and Ameliorates Ovariectomy-Induced Bone Loss

**DOI:** 10.3389/fphar.2020.621110

**Published:** 2021-01-15

**Authors:** Dahu Qi, Hui Liu, Xuying Sun, Danni Luo, Meipeng Zhu, Tenghui Tao, Chenghao Gao, Chuankun Zhou, Wei Zhou, Jun Xiao

**Affiliations:** ^1^ Department of Orthopedics, Tongji Hospital, Tongji Medical College, Huazhong University of Science and Technology, Wuhan, China; ^2^ Department of Orthopedics, Zhongnan Hospital of Wuhan University, Wuhan, China; ^3^ Institute of Hepatobiliary Diseases, Transplant Center, Hubei Key Laboratory of Medical Technology on Transplantation, Zhongnan Hospital of Wuhan University, Wuhan, China

**Keywords:** pristimerin, osteoclastogenesis, MAPK, NF-κB, Nrf2

## Abstract

Osteoporosis is characterized by bone loss and destruction of trabecular architecture, which greatly increases the burden on the healthcare system. Excessive activation of osteoclasts is an important cause of osteoporosis, and suppression of osteoclastogenesis is helpful for the treatment of osteoporosis. Pristimerin, a natural compound, possesses numerous pharmacological effects via inactivating the NF-κB and MAPK pathways, which are closely related to osteoclastogenesis process. However, the relationship between Pristimerin and osteoclastogenesis requires further investigation. In this research, we examined the effect of Pristimerin on osteoclastogenesis and investigated the related mechanisms. Our results showed Pristimerin inhibited RANKL-induced osteoclast differentiation and osteoclastic bone resorption *in vitro*, with decreased expression of osteoclastogenesis-related markers including c-Fos, NFATc1, TRAP, Cathepsin K, and MMP-9 at both mRNA and protein levels. Furthermore, Pristimerin suppressed NF-κB and MAPK signaling pathways, reduced reactive oxygen species (ROS) production and activated the nuclear factor erythroid 2-related factor 2/heme oxygenase 1 (Nrf2/HO-1) signaling during osteoclastogenesis. Our *in vivo* experiments showed that Pristimerin remarkably ameliorated ovariectomy-induced bone loss, reduced serum levels of TNF-α, IL-1β, IL-6, and RANKL, and increased serum level of osteoprotegerin (OPG). Therefore, our research indicated that Pristimerin is a potential chemical for the treatment of osteoporosis.

## Introduction

Bone, a dynamic organ, is in a constant remodeling to maintain homeostasis. Osteoblast and osteoclast are the two main cells involved in bone remodeling, which are responsible for bone formation and bone resorption, respectively (Siddiqui and Partridge, 2016). A disbalance in bone remodeling causes numerous bone metabolism diseases, such as osteoporosis, osteopetrosis, Paget’s disease and rheumatoid arthritis (Roodman and Windle, 2005; Feng and McDonald, 2011; Baum and Gravallese, 2016).

In recent years, osteoporosis has become one of the most important public health problems, which is characterized by reduced bone density and destruction of trabecular architecture, and can easily lead to fractures and body pain (Ensrud and Crandall, 2017). As bone resorption mainly depends on osteoclasts, excessive activation of osteoclasts is the principal cause of osteoporosis (Charles and Aliprantis, 2014).

Osteoclasts are multinucleated giant cells originated from precursor cells of monocyte/macrophage haematopoietic lineage (Ono and Nakashima, 2018). Macrophage colony-stimulating factor (M-CSF) and receptor activator of nuclear factor-κB ligand (RANKL) are the two vital factors for the maturation of osteoclasts (Boyle et al., 2003). The combination of M-CSF and its receptor c-Fms can regulate proliferation, differentiation and survival of osteoclasts and their precursors, as well as induce the expression of RANK on these cells (Boyce, 2013). RANKL associates with RANK on the surface of osteoclast precursors, activates multiple downstream signaling pathways, including NF-κB, MAPK and activator protein-1 (Nakashima and Takayanagi, 2011), which are most important pathways involved in osteoclastogenesis. Furthermore, pro-inflammatory cytokines, ROS production and Nrf2/HO-1 signals are also strongly associated with osteoclastogenesis (Agidigbi and Kim, 2019; Coury et al., 2019; Sun et al., 2020).

Pristimerin, a natural triterpenoid compound, isolated from the traditional Chinese herbs *Celastraceae* and *Hippocrateaceae*, has shown numerous biological and pharmacological properties, such as anti-inflammatory (Yadav et al., 2010), anti-oxidative (Dos Santos et al., 2010; Hui et al., 2014), anti-tumor (Li et al., 2019), anti-malarial (Figueiredo et al., 1998) and anti-fungal effects (Luo et al., 2005; Gullo et al., 2012). Many previous studies have confirmed that Pristimerin exerts its pharmacological effects through inactivating NF-κB and MAPK signaling (Deeb et al., 2014; Yousef et al., 2018; Xie et al., 2019), which are similar pathways involved in osteoclastogenesis. However, the role of Pristimerin on osteoclastogenesis need further investigation.

Therefore, we hypothesized that Pristemerin may inhibit osteoclastogenesis through the anti-inflammatory mechanism. To test our hypothesis, we investigated the effect of Pristimerin on osteoclastogenesis both *in vitro* and *in vivo*, and explored the underlying mechanisms.

## Materials and Methods

### Reagents and Antibodies

Pristimerin (HPLC ≥ 98%) was bought from Sigma-Aldrich (St.Louis, MO, USA), the compound was dissolved in DMSO, and stored in a refrigerator at −80°C for later use. α-MEM medium and cell counting kit-8 (CCK-8) kit were bought from Boster (Wuhan, China). Specific antibodies against Nrf2, NFATc1, HO-1, MMP-9, NQO-1, c-Fos, tartrate-resistant acid phosphatase (TRAP), GAPDH, and Cathepsin K (CTSK) were obtained from Proteintech Group (Wuhan, China). The Rhodamine-conjugated phalloidin, TRAP staining kit, and DAPI were bought from Sigma-Aldrich (St.Louis, MO, USA). Specific antibodies against IKKβ, p-JNK, JNK, p-ERK, ERK, p-P38, P38, p-IκBα, IκBα, p-P65, P65, and p-IKKα/β were bought from Cell Signaling Technology (Beverly, MA, USA). Osteo Assay Surface was bought from Corning Incorporated Life Science (Corning, NY, USA). M-CSF and RANKL were bought from R&D Systems (Minneapolis, MN, USA).

### Bone Marrow-Derived Macrophages Culture and Cytotoxicity Assay

We isolated bone marrow-derived macrophages (BMMs) from the femoral and tibial bone marrow of 8-week-old male C57BL/6 mice as showed previously (Guan et al., 2015). BMMs were cultured in α-MEM medium supplemented with 10% fetal bovine serum, streptomycin (100 ug/mL), penicillin (100 U/mL) and M-CSF (30 ng/ml) at 37°C in an incubator. CCK-8 kit was used to test the cytotoxicity of Pristimerin in BMMs. Briefly, 2.4 × 10^3^ BMMs were plated onto 96-well plates each well, and were incubated overnight, then the BMMs were exposed to various concentrations (0, 5, 10, 25, 50, and 75 nM) of Pristimerin for 1, 3, and 5 days. Medium was changed daily. At the end of culture, 10 ul CCK-8 solution was added to each well and incubated at 37°C for 1 h at dark. Finally, the optical density (OD) was measured at 450 nm with a plate reader.

### TRAP Staining Assay

We used TRAP staining to assess the effect of Pristimerin on osteoclast formation *in vitro*. Briefly, 1.8 × 10^4^ BMMs were plated onto 96-well plates per well overnight. Then multiple concentrations (0, 5, 10, 25, 50, and 75 nM) of Pristimerin were added to the medium supplemented with RANKL (100 ng/ml) for additional 6–7 days, with medium changed daily. When BMMs were observed to fuse into osteoclasts under a light microscope, the cells were rinsed for 3 times and fixed in 4% paraformaldehyde for 20 min, then the cells were stained using the TRAP kit (Sigma) according to the instructions. Multinucleated (>3 nuclei) TRAP-positive cells were identified as osteoclasts.

### Bone Pit and Actin Ring Formation Assay

BMMs were seeded on 0.2% collagen-gel pre-coated 24-well plates and stimulated with RANKL (100 ng/ml) for 6–7 days. Type I collagenase was used to obtain the mature osteoclasts and the cells were plated onto Corning Osteo Assay surface, then the cells were treated with different concentrations (0, 10, 25, 50, and 75 nM) of Pristimerin with or without RANKL (100 ng/ml) supplement for additional 3 days. As for pit formation, the plates were soaked in 5% sodium hypochlorite for 5 min and washed with pure water, then the bone resorption areas were photographed for further quantitative analysis. For actin ring formation, 4% paraformaldehyde was used to fix the cells for 20 min, 0.2% Triton X-100 was used to break the cell membranes for 10 min. Then the Phalloidin and DAPI were applied to stain the cells respectively. Finally, a fluorescence microscope was used to take the actin ring images.

### Bone Marrow Stem Cells Isolation and Osteogenic Differentiation Assay

BMSCs were isolated from the femurs and tibias of 5 weeks old SD rats as shown previously (Watanabe et al., 2018), and cultured with DMEM/F12 medium until confluent. Then the BMSCs were digested and seeded on 24-well plates at a density of 1.5 × 10^4^ cells/well. For osteogenic differentiation, the BMSCs were cultured in osteogenic medium (a-MEM medium containing 50 mM Ascorbic acid, 10 nM dexamethasone and 10 mM β-glycerol phosphate) and the medium was replaced every 2 days. ALP staining was performed at day 7 after differentiation using the BCIP/NBT Alkaline phosphatase Color Development Kit (Beyotime, China). Alizarin Red staining was performed at day 14 after differentiation using the Alizarin Red (Cyagen Biosciences) according to the manufacturers’ protocols.

### Quantitative Real-Time Polymerase Chain Reaction

Total RNA of cultured cells were extracted with TRIzol reagent (Invitrogen, Carlsbad, CA, USA). 1μg RNA was used to synthesize the complementary DNA (cDNA) using the ReverTra Ace qPCR RT Master Mix kit (Toyobo, Osaka, Japan). TB Green Fast qPCR Mix (Takara, Japan) was used to perform RT-PCR. The primer sequences used were as follows (sequences 5′–3′, sense and antisense): *TRAP*: GAT​GCC​AGC​GAC​AAG​AGG​TT and CAT​ACC​AGG​GGA​TGT​TGC​GAA; MMP-9: CTG​GAC​AGC​CAG​ACA​CTA​AAG and CTC​GCG​GCA​AGT​CTT​CAG​AG; NFATc1: TCT​TCC​GAG​TTC​ACA​TCC​C and GAC​AGC​ACC​ATC​TTC​TTC​C; CTSK: GAA​GAA​GAC​TCA​CCA​GAA​GCA​G and TCC​AGG​TTA​TGG​GCA​GAG​ATT; GAPDH (mouse): AAC​GAC​CCC​TTC​ATT​GAC​CTC and CCT​TGA​CTG​CCG​TTG​AAC​T; GAPDH (rat): GGT​GGA​CCT​CAT​GGC​CTA​CA and CTC​TCT​TGC​TCT​CAG​TAT​CCT​TGC​T; ALP: GCA​CAA​CAT​CAA​GGA​CAT​CG and TCA​GTT​CTG​TTC​TTG​GGG​TAC​AT; RUNX2: GGG​ACC​GAC​ACA​GCC​ATA​TA and TCT​TAG​GGT​CTC​GGA​GGG​AA; OCN: GCC​CTG​ACT​GCA​TTC​TGC​CTC​T and TCA​CCA​CCT​TAC​TGC​CCT​CCT​G; OPN: CCA​GCC​AAG​GAC​CAA​CTA​CA and GCT​GGC​AGT​GAA​GGA​CTC​AT.

### Western Blot Analysis

RIPA lysis buffer was used to extract total proteins from cultured BMMs. 15 μg proteins were separated using 10% SDS-PAGE gel electrophoresis, then the proteins were transferred to PVDF membranes (Millipore, Billerica, MA, USA). 5% BSA solutions was used to block the membranes for 1 h at room temperature, then the membranes were incubated with specific primary antibodies for 16 h at 4°C, with a dilution ratio of 1:1,000. Membranes were washed and incubated with the secondary antibodies for another 1 h. Finally, electrochemiluminescence reagents (Thermo Fisher Scientific) were used to acquire the images and the band gray values were analyzed with Image Lab 5.1 software (Bio-Rad, Hercules, CA).

### Measurement of Reactive Oxygen Species Expression

DCFH-DA (Beyotime, Shanghai, China) was used to measure the levels of ROS in BMMs. Briefly, BMMs were stimulated with or without RANKL (100 ng/ml), and were exposed to various concentrations (0, 25, 50, and 75 nM) of Pristimerin for 24 h. Then DCFH-DA (10 μM) was added to each well and incubated at 37°C at dark for 25 min. The cells were washed twice and a fluorescence microscope was used to observe the intracellular ROS level.

### Animals and Ovariectomized Mouse Model

An ovariectomized (OVX) osteoporosis model was built to assess the role of Pristimerin on bone loss *in vivo*. All animal experiments were performed on the basis of the recommendations of Animal Experimentation Guidelines, the Ethics Committee on Animal Experimentation of Tongji Medical College, Huazhong University of Science and Technology (Wuhan, China). Thirty 8-week-old female C57BL/6 mice were obtained from the Experimental Animal Center of Tongji Hospital (Wuhan, China) and were randomly divided into three groups (n = 10): The sham + VEH (Sham group treated with vehicle), the OVX + VEH (OVX group treated with vehicle), and the OVX + Pristimerin (OVX group treated with Pristimerin). All mice were anesthetized and the bilateral ovaries were removed to induce osteoporosis except the sham group. Three days after operation, the mice were intraperitoneally injected with Pristimerin (1 mg/kg) or vehicle (DMSO) for 5 days per week. 8 weeks later, all mice were sacrificed, the femurs and sera were collected for following experiments.

### μCT Scaning

After removal of the soft tissues, the femurs were fixed in 4% paraformaldehyde for 3 days. µCT (Scanco Medical, Bassersdorf, Switzerland) was applied to scan the metaphysical regions of left femurs, the voltage of the source was set to 70 kV, the current of the source was set to 110 μA, and the isotropic resolution was set to 9 μm. We used built-in μCT software to reconstruct three-dimensional images of the femurs and analyzed the bone structural parameters, such as bone volume/tissue volume (BV/TV), trabecular separation (Tb.Sp), trabecular number (Tb.N), and trabecular thickness (Tb.Th).

### Histological Analyses

All right femurs were soaked in 10% EDTA for 2 weeks, then the tissues were embedded in paraffin, and were cut at 5 μm using a microtome. H&E and TRAP staining were performed to observe the bone structure or the osteoclasts.

### Measurement of Serum Inflammatory Factors

The sera of all the mice were collected. The levels of TNF-α, IL-1β, IL-6, OPG, and RANKL in serum were detected using ELISA kits based on the manufacturer's protocols.

### Statistical Analyses

The data were showed as means ± SD, and all experiments were repeated at least three times. Student's t test was used to examine the statistical significance between the two groups, while one-way ANOVA was used for multiple comparisons. A *p*-value < 0.05 was considered as significant, all statistical analysis was performed with Prism 8 software (GraphPad Software Inc., La Jolla, CA, USA).

## Results

### Pristimerin Suppresses Receptor Activator of Nuclear Factor-κB Ligand-Induced Osteoclast Differentiation *in vitro*

**FIGURE 1:**
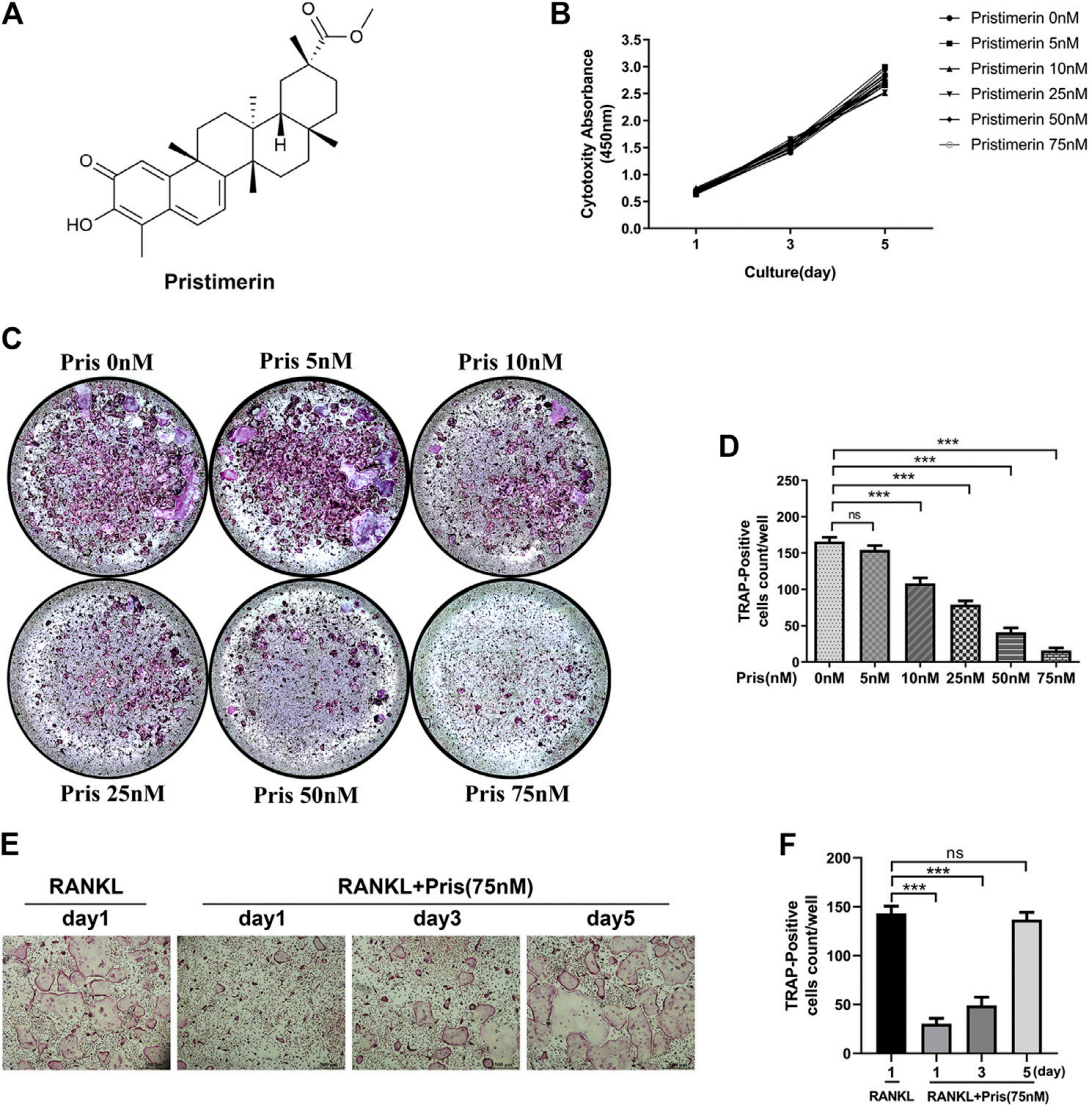
Pristimrin suppresses RANKL-induced osteoclast formation *in vitro* **(A)** Chemical formula of Pristimerin **(B)** Pristimerin concentrations lower than 75 nM did not have cytotoxic effect on BMMs. BMMs were treated with various concentrations (0, 5, 10, 25, 50, and 75 nM) of Pristimerin for 1, 3, and 5 days, and the cytotoxicity was evaluated using a CCK-8 kit **(C and D)** Pristimerin suppressed RANKL-induced osteoclast formation. BMMs were treated with various concentrations (0, 5, 10, 25, 50, and 75 M) of Pristimerin in the presence of RANKL (100 ng/ml) for 6–7 days. The number of osteoclasts was counted using ImageJ **(E and F)** Pristimerin suppressed osteoclast formation at early stage (1–3 days), but not late stage (5–7 days). BMMs were treated with Pristimerin (75 nM) at day 1, 3, and 5 after RANKL-induced osteoclastogenesis. The TRAP staining was performed 6–7 days after RANKL stimulation. The data were obtained in three independent experiments and were shown as mean ± SD (*p < 0.05, **p < 0.01, and ***p < 0.001).

Pristimerin (Pris), a natural compound, possesses numerous pharmacological effects, its chemical formula is showed in Figure 1A. First, we used CCK-8 kit to measure the cytotoxicity of Pristimerin in BMMs. The results demonstrated that there was no obvious effect on the cytotoxicity of BMMs at 1, 3, 5 days after Pristimerin incubation (Figure 1B). To test the role of Pristimerin on osteoclasts formation, BMMs were treated with increasing concentrations (0, 5, 10, 25, 50, and 75 nM) of Pristimerin for 6–7 days in the presence of RANKL (100 ng/ml). We found that RANKL-induced osteoclasts formation was obviously suppressed by Pristimerin in a concentration-dependent manner (Figure 1C,D). In addition, to identify Pristimerin inhibits osteoclastogenesis at which stage, BMMs were treated with Pristimerin (75 nM) at day 1, 3, and 5 after osteoclastogenesis. The results indicated that Pristimerin inhibited osteoclasts formation at early stage (1–3 days), but not late stage (5–7 days) (Figure 1E,F). Therefore, Pristimerin significantly suppressed RANKL-induced osteoclast formation without cytotoxic effect on BMMs. To explore the role of Pristimerin on osteogenesis in BMSCs, we also performed ALP staining at day 7 and Alizarin Red staining at day 14 after differentiation, the results indicated that there was no obvious effect on osteogenesis in BMSCs after Pristimerin treatment (Supplementary Figure S1A,B).

### Pristimerin Impairs Osteoclastic Bone Resorption *in vitro*

**FIGURE 2:**
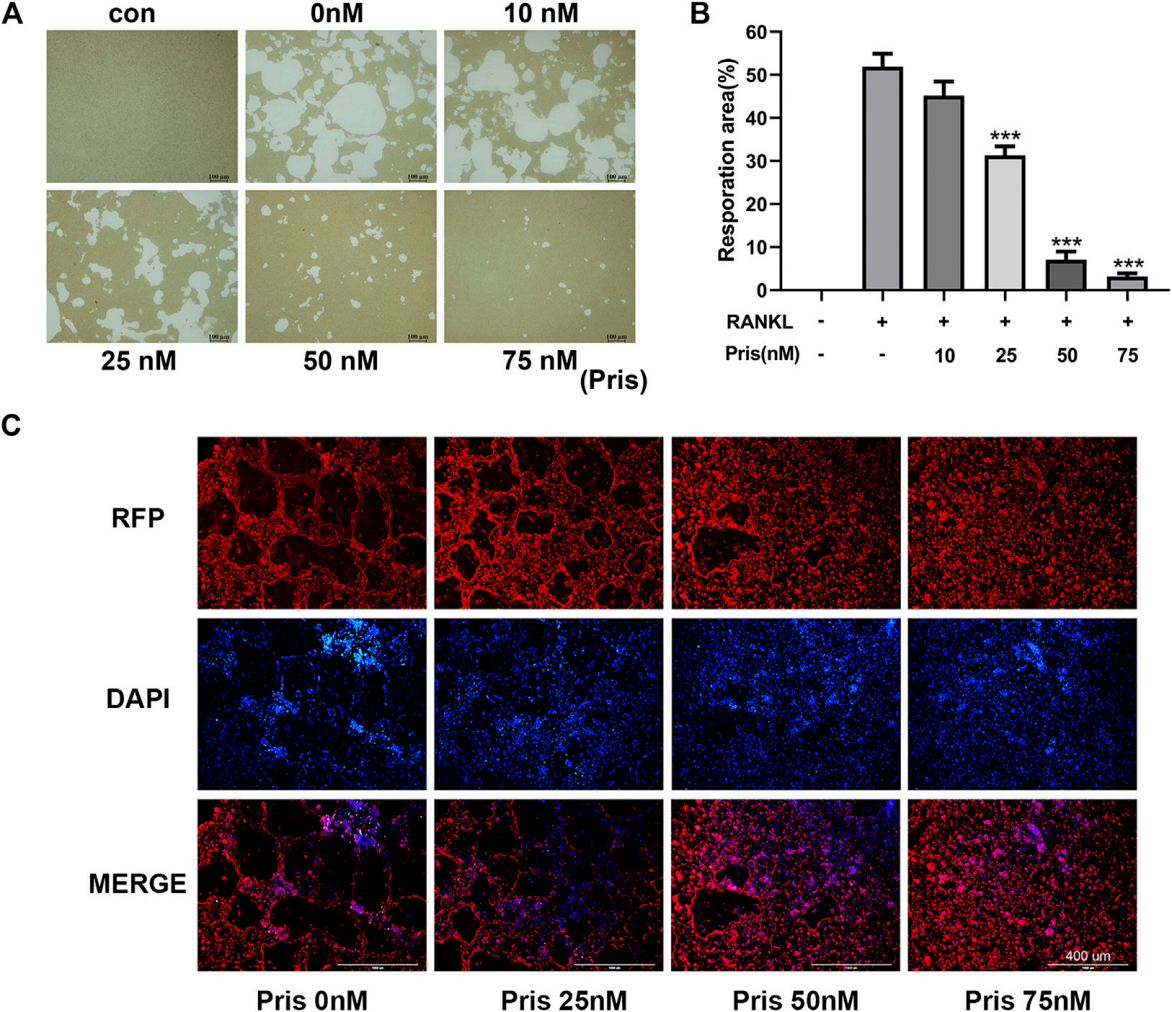
Pristimerin impairs RANKL-induced osteoclastic bone resorption *in vitro* **(A)** Pristimerin remarkably inhibited bone pit formation. BMMs were stimulated with RANKL to obtain mature osteoclasts and the cells were plated onto Corning osteo assay surface, then the cells were treated with various concentrations (0, 10, 25, 50, and 75 nM) of Pristimerin with or without RANKL (100 ng/ml) supplement for another 3 days. All resorption areas were photographed by a microscope **(B)** Bone resorption areas were assessed with ImageJ software **(C)** Pristimerin significantly inhibited actin ring formation. The osteoclasts were stained with phalloidine and DAPI respectively, then photographed by a fluorescence microscope. The data were obtained in three independent experiments and were shown as mean ± SD (*p < 0.05, **p < 0.01, and ***p < 0.001, vs. RANKL + 0 nM Pristimerin group).

To explore the effect of Pristimerin on the osteoclast function, pit formation and actin ring formation experiments were performed. The results revealed that the bone resorption area was remarkably reduced by Pristimerin in a dose-dependent manner (Figure 2A,B). Actin ring formation is vital and essential for osteoclast attachment and bone resorption (Wilson et al., 2009). In our study, the actin ring formation was also suppressed after the treatment of Pristimerin (Figure 2C). Therefore, Pristimerin significantly inhibited osteoclastic bone resorption *in vitro*.

### Pristimerin Inhibits Receptor Activator of Nuclear Factor-κB Ligand-Induced Osteoclastogenesis-Related Markers Expression *in vitro*

**FIGURE 3:**
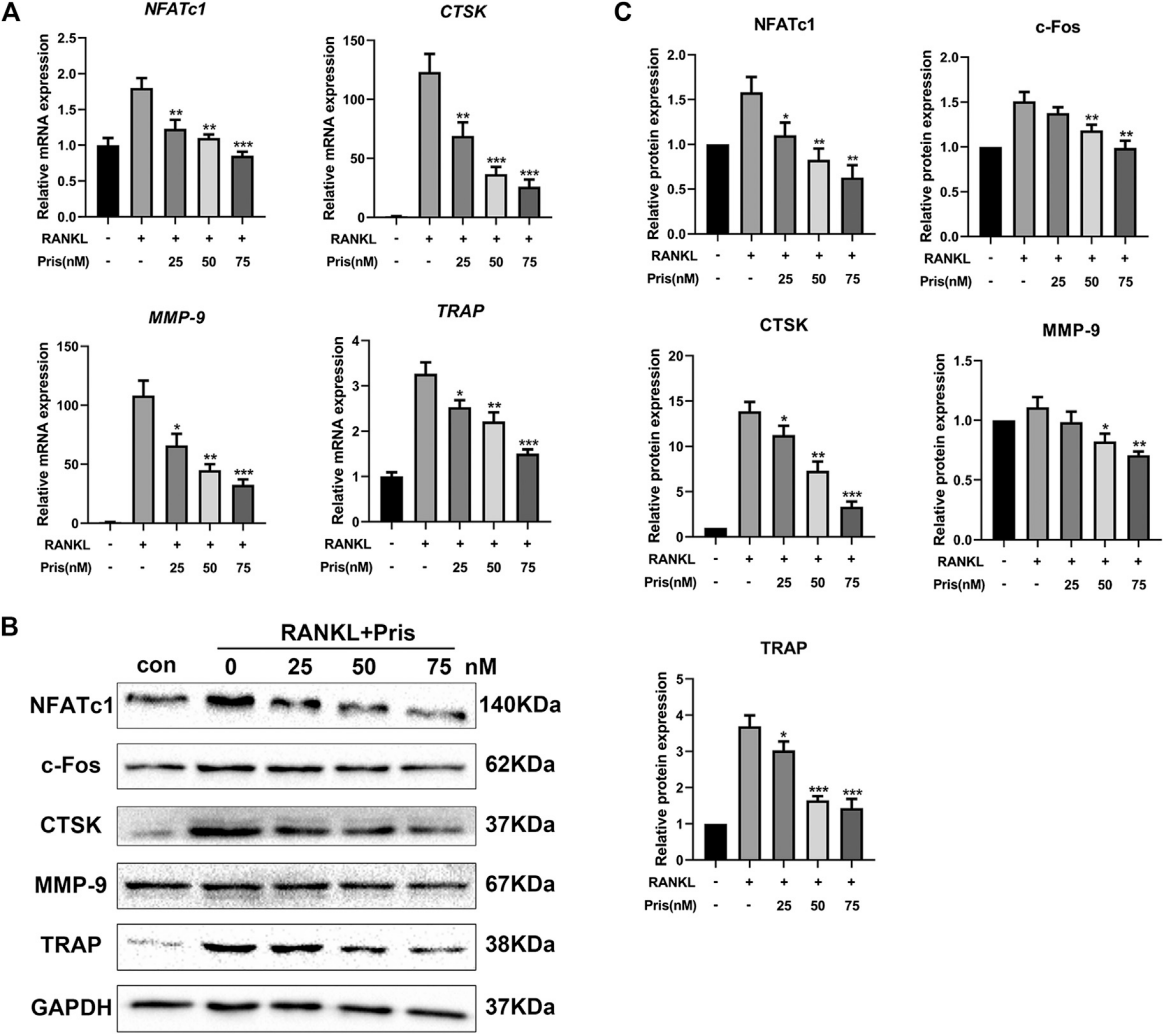
Pristimerin inhibits RANKL-induced osteoclastogenesis-related markers expression *in vitro* **(A)** Pristimerin suppressed mRNA expression levels of *NFATc1*, *CTSK*, *MMP-9*, and *TRAP*. BMMs were treated with virous concentrations (0, 25, 50, and 75 nM) of Pristimerin in the presence or absence of RANKL (100 ng/ml) for 3 days, total RNA was extracted and analyzed using RT-PCR **(B and C)** Pristimerin suppressed protein expression levels of NFATc1, c-Fos, CTSK, TRAP, and MMP-9. The cells were processed as above, the BMMs were cultured for 5 days, total proteins were collected and then analyzed using western blot. The data were obtained in three independent experiments and were shown as mean ± SD (*p < 0.05, **p < 0.01, and ***p < 0.001, vs. RANKL + 0 nM Pristimerin group).

To further explore the role of Pristimerin on osteoclastogenesis in BMMs, the mRNA and protein expression levels of osteoclastogenesis-related genes were detected, including *TRAP*, *Cathepsin K*, *NFATc1*, *MMP-9*, and *c-Fos*. These markers play a very important part in osteoclast differentiation and function (Ono and Nakashima, 2018). The results indicated that Pristimerin significantly suppressed the osteoclastogenesis-related markers expression in BMMs in a concentration-dependent manner (Figure 3). To explore the role of Pristimerin on osteogenesis in BMSCs, the mRNA expression levels of osteogenesis-related genes including *ALP*, *RUNX2*, *OCN*, *OPN* were also detected, the results indicated that there was no obvious effect on osteogenesis after Pristimerin treatment for 7 days (Supplementary Figure 1C).

### Pristimerin Inhibits NF-κB and MAPK Pathways

**FIGURE 4:**
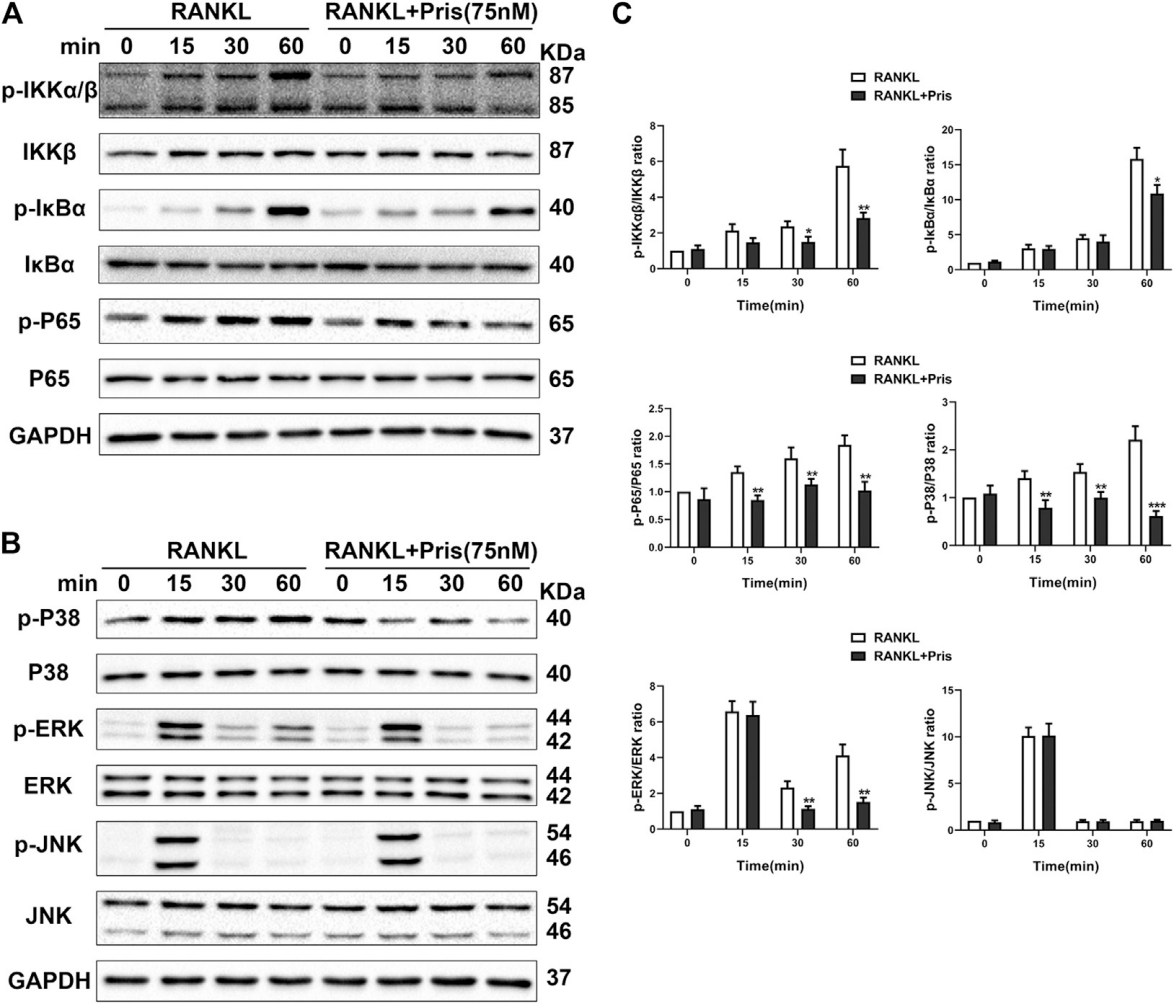
Pristimerin suppresses RANKL-induced activation of NF-κB and MAPK pathways. BMMs were pretreated with or without Pristimerin (75 nM) in 3% FBS for 12h, then stimulated with RANKL (100 ng/ml) in the presence or absence of Pristimerin (75 nM) for indicated time points. Total and phosphorylated proteins were measured using western blot analysis **(A and C)** Pristimerin inhibited NF-κB pathway activation via reducing the phosphorylation of P65, IKKα/β, and IκBα **(B and C)** Pristimerin inhibited MAPK pathway activation via suppressing the phosphorylation of P38 and ERK but not JNK. The data were obtained in three independent experiments and were shown as mean ± SD (*p < 0.05, **p < 0.01, and ***p < 0.001, vs. the RANKL group).

To uncover the molecular mechanism underlying the inhibitory effect of Pristimerin on osteoclastogenesis, we examined the expression level of proteins involved in NF-κB and MAPK pathways. Briefly, BMMs were starved with 3% FBS in the presence of Pristimerin (75 nM) or not for 12 h, then RANKL (100 ng/ml) was added to the medium for 0, 15, 30 and 60 min. Western blot results showed Pristimerin decreased the phosphorylation of IκBα, IKKα/β, and P65 (Figure 4A,C), the phosphorylation of P38 and ERK also decreased, but JNK remained unchanged (Figure 4B,C), indicating both NF-κB and MAPK pathways were suppressed by Pristimerin.

### Pristimerin Suppresses Receptor Activator of Nuclear Factor-κB Ligand-Induced Reactive Oxygen Species Production and Activates the Nrf2/HO-1 Signals

**FIGURE 5:**
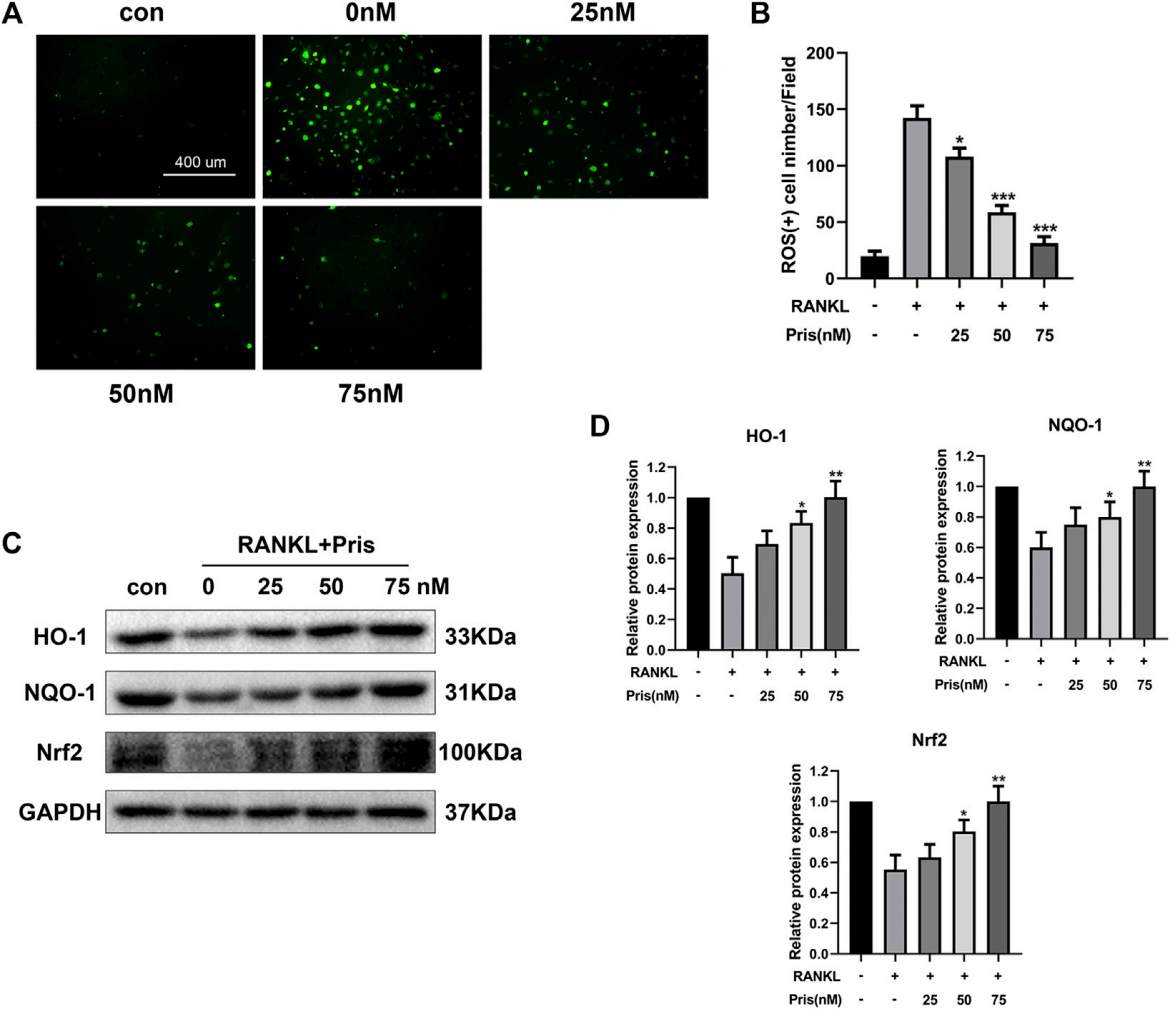
Pristimerin suppresses RANKL-induced ROS production and activates Nrf2/HO-1 signaling **(A)** Pristimerin suppressed RANKL-induced ROS expression in BMMs. BMMs were treated with various concentrations (0, 25, 50, and 75 nM) of Pristimerin in the presence or absence of RANKL (100 ng/ml) for 24 h, then incubated with diluted DCFH-DA (10 μM) for 25 min at dark. Finally, a fluorescence microscope was used to observe the ROS-positive cells **(B)** The number of ROS-positive cells was counted using ImageJ **(C and D)** Pristimerin treatment increased Nrf2, NQO-1, and HO-1 expression levels. The data were obtained in three independent experiments and were shown as mean ± SD (*p < 0.05, **p < 0.01, and ***p < 0.001, vs. RANKL + 0 nM Pristimerin group).

Overproduction of ROS promotes osteoclast formation and plays an important part in osteoclastogenesis (Agidigbi and Kim, 2019). The activation of Nrf2/HO-1 signals could induce the expression of various antioxidants and protect cells from oxidative stress (Bellezza et al., 2018). Therefore, the role of Pristimerin on oxidative stress in BMMs was investigated. The DCFH-DA staining assay showed that Pristimerin treatment significantly reduced the number of ROS-positive cells induced by RANKL stimulation (Figure 5A,B). We also tested the effect of Pristimerin on Nrf2/HO-1 signaling. BMMs were treated with different concentrations (0, 25, 50, and 75 nM) of Pristimerin in the presence or absence of RANKL (100 ng/ml) for 3 days, then the total proteins were extracted for western blot. The results showed that the protein levels of Nrf2, HO-1, and NQO-1 decreased after RANKL stimulation, but were significantly increased after various concentrations (0, 25, 50, and 75 nM) of Pristimerin incubation in BMMs (Figure 5C,D). Taken together, Pristimerin decreased the ROS production and activated Nrf2/HO-1 signaling.

### Pristimerin Ameliorates Ovariectomized-Induced Bone Loss *in vivo*

**FIGURE 6:**
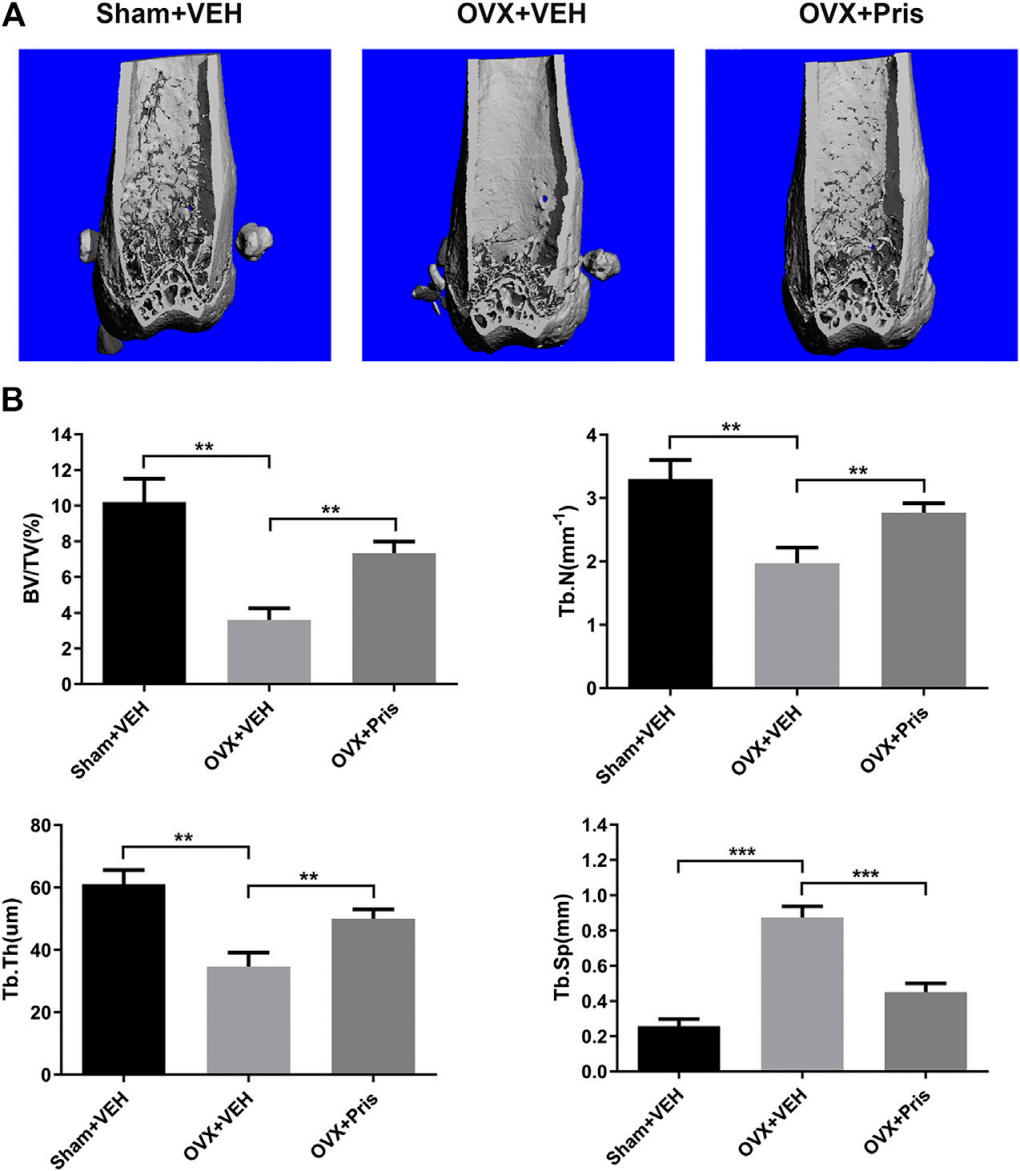
Pristimerin ameliorates OVX-induced bone loss *in vivo* **(A)** Representative three-dimensional reconstruction images of distal femurs of Sham + VEH (sham group treated with vehicle), OVX + VEH (OVX group treated with vehicle), and OVX + Pristimerin (OVX group treated with Pristimerin). The results showed that Pristimerin treatment could ameliorate OVX-induced bone loss **(B)** Statistical graphs of bone parameters BV/TV, Tb.Sp, Tb.Th, and Tb.N. Data were described as mean ± SD (n = 10) (*p < 0.05, **p < 0.01, and ***p < 0.001, vs. OVX + VEH group).

To test the function of Pristimerin *in vivo*, an OVX-induced osteoporosis model was created to evaluate the potential protective effect of Pristimerin on osteoporosis. Mice were injected with Pristimeirn (1 mg/kg) or vehicle intraperitoneally for 8 weeks. After sacrificing, the left femurs of mice were scanned and analyzed using μCT. The results indicated that the trabecular bone mass of mice in OVX group reduced significantly compared with the sham group, while treatment with Pristimerin dramatically rescued the OVX-induced bone loss (Figure 6A), as also confirmed by the bone parameters. Ovariectomy decreased BV/TV, Tb.N, and Tb.Th but increased Tb.Sp in mice, and this were reversed by Pristimerin treatment (Figure 6B).

**FIGURE 7:**
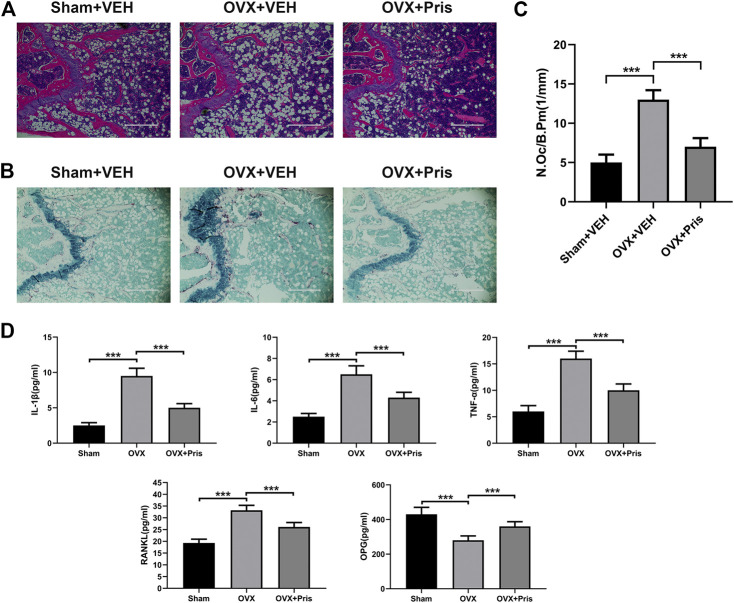
Pristimerin improves histomorphological changes of bone tissue in OVX mice **(A)** Representative H&E staining sections from each group **(B)** Representative TRAP staining sections from each group **(C)** The osteoclasts on the surface of trabecular bone was counted using ImageJ **(D)** Pristimerin increased the serum level of OPG and decreased the serum levels of TNF-α, IL-1β, IL-6, and RANKL. These cytokines were measured using ELISA kits. Date were described as mean ± SD (n = 10) (*p < 0.05, **p < 0.01, and ***p < 0.001, vs. OVX + VEH group).

The histological examinations were also performed to identify the effect of Pristimerin on bone loss. H&E staining showed that Pristimerin treatment significantly ameliorated the trabecular bone loss caused by ovariectomy (Figure 7A). TRAP staining indicated that Pristimerin application obviously reduced the number of osteoclasts on the trabecular bone surface near distal femur growth plate, compared with the OVX mice (Figure 7B,C).

In addition, we tested the levels of TNF-α, IL-1β, IL-6, OPG, and RANKL in the sera of mice using ELISA kits. We found that compared to the sham group, TNF-α, IL-1β, IL-6, and RANKL levels increased in the OVX group, and all these inflammatory factors were reversed in OVX treated with Pristimerin group, which confirmed the anti-inflammatory effect of Pristimerin *in vivo*. Moreover, Pristimerin treatment increased OPG level compared to OVX group (Figure 7D).

## Discussion

**FIGURE 8:**
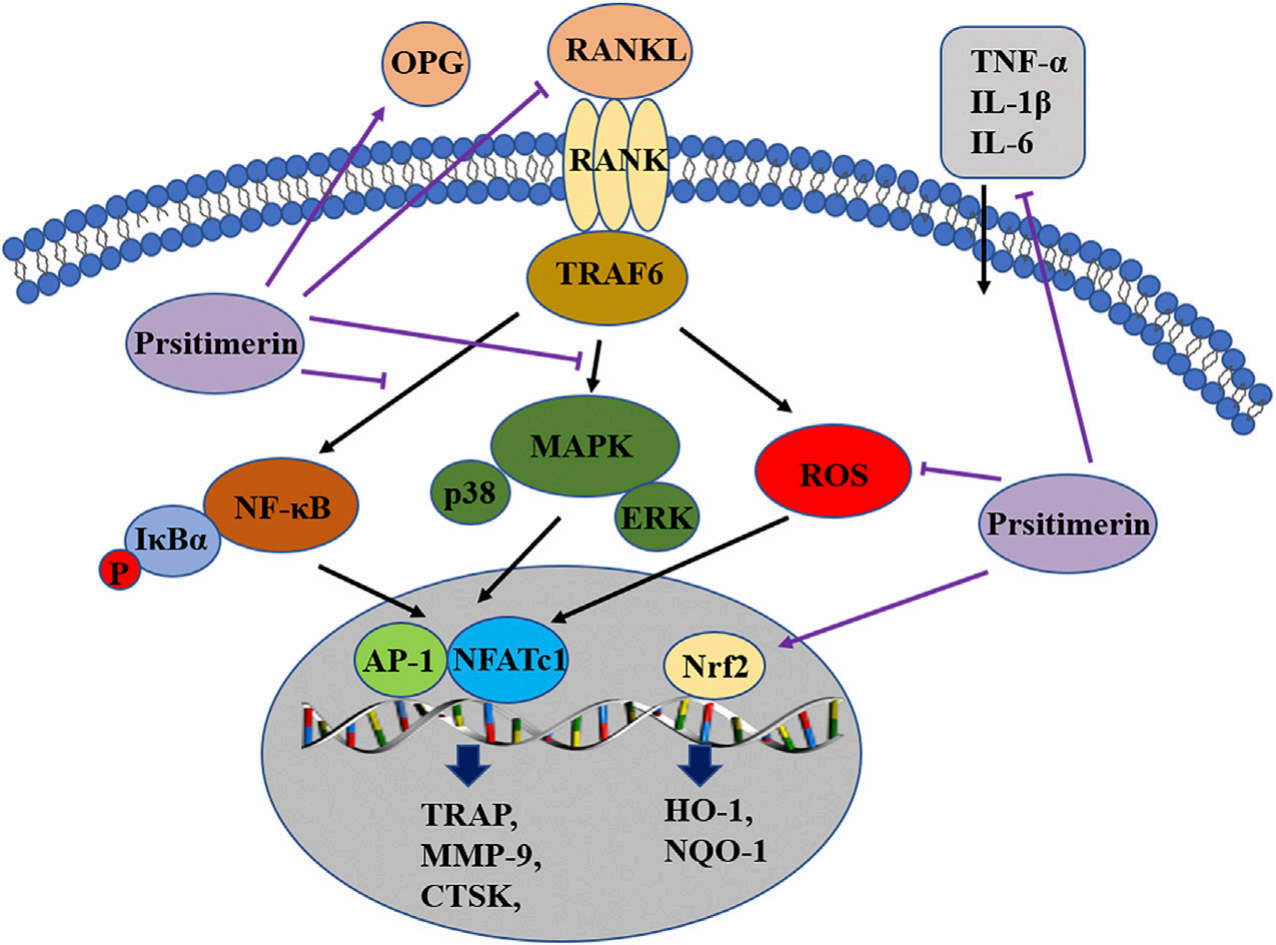
Pristimerin suppresses osteoclastogenesis through multiple pathways. Pristimerin inhibited the activation of NF-κB and MAPK pathways, and activated the Nrf2/HO-1 signaling. Pristimerin decreased RANKL-induced ROS production. Pristimerin suppressed the production of pro-inflammatory factors TNF-α, IL-1β, IL-6, and RANKL, but increased OPG level in the serum.

In the current study, the effect of Pristimerin on osteoclastogenesis was revealed *in vitro* and *in vivo*. Our results indicated that Pristimerin inhibited RANKL-induced osteoclast differentiation and function, reduced the expression of osteoclastogenesis-specific markers, including NFATc1, c-Fos, TRAP, CTSK, and MMP-9. The ovariectomized mice were selected for our *in vivo* experiments, which showed that Pristimerin reduced the levels of inflammatory factors in mice and alleviated the bone loss caused by ovariectomy. We also found Pristimerin inactivated NF-κB and MAPK pathways and activated Nrf2/HO-1 signaling, which might be the potential molecular mechanisms for its role on osteoclastogenesis (Figure 8). Osteoclastic bone resorption and osteoblastic bone formation maintain bone homeostasis together (Feng and McDonald, 2011), thus some experiments on osteogenesis in BMSCs were performed as a supplement. In the current study, we found there was no obvious effect on osteogenesis after Pristimerin treatment in BMSCs.

Current evidence suggests that RANKL/RANK/OPG axis plays a major part in osteoclastogenesis (Walsh and Choi, 2014). OPG associates with RANKL and functions as a decoy receptor, thus inhibiting the differentiation of osteoclasts (Baud’huin et al., 2007). RANKL binds to RANK, results in the recruitment of TRAF6 in the cytoplasm, then triggers the downstream NF-κB and MAPK signaling, which subsequently activates transcription factors NFATc1 and c-Fos, thus enhances the expression of osteoclastogenesis-related markers, eventually leads to the differentiation and maturation of osteoclasts (Takayanagi et al., 2002; Wada et al., 2006). Recent literatures have clearly demonstrated that Pristimerin could suppress the activation of MAPK and NF-κB signaling (Tu et al., 2018). One study found that Pristimerin exerted anti-inflammatory effects via blocking the NF-κB and MAPK signals in murine macrophage RAW264.7 cells (Kim et al., 2013). Another study showed that Pristimerin could suppress LPS-induced neurotoxicity via inhibiting NF-κB and JNK/AP-1 pathways in BV-2 microglia cells (Hui et al., 2018). In our study, Pristimerin suppressed the phosphorylation of P65, IκBα, IKKα/β, P38 and ERK, thus blocked the activation of NF-κB and MAPK, and this might be the molecular basis by which Pristimerin inhibits osteoclastogenesis.

ROS have been shown to mediate the toxicity but also function as signaling molecules (D’Autreaux and Toledano, 2007). The excessive production of ROS may be responsible for various pathological conditions, such as tumors (Prasad et al., 2017), inflammation (Mittal et al., 2014) and osteoporosis (Agidigbi and Kim, 2019). Nuclear factor erythroid-related factor 2 (Nrf2) is a vital regulator of oxidative stress response, it controls the expression of downstream antioxidant enzymes such as HO-1 and NQO-1 (Tonelli et al., 2018). Nrf2 activation could counteract RANKL-induced ROS production and further suppress osteoclastogenesis (Sun et al., 2015; Liu et al., 2019). One study reported that Pristimerin could protect against DOX-induced cardiotoxicity due to activation of Nrf2 and inhibition of MAPK signaling (El-Agamy et al., 2019). Another study indicated that Pristimerin possessed protective effects against autoimmune hepatitis via the activation of Nrf2/HO-1 pathway, thus exerted its anti-oxidative, anti-inflammatory properties (El-Agamy et al., 2018). In our study, Pristimerin effectively inhibited the generation of ROS induced by RANKL and increased the expression of Nrf2, HO-1, and NQO-1 in BMMs, this could also be one of the mechanisms by which Pristimerin inhibits osteoclastogenesis.

Inflammation is also closely related to osteoclastogenesis (Adamopoulos, 2018). Studies have implicated that postmenopausal osteoporosis is partially mediated by the release of pro-inflammatory factors due to estrogen withdrawal, such as IL-6, TNF-α, and IL-1 (Mundy, 2007). Overproduction of pro-inflammatory cytokines is also associated with some inflammatory bone destruction diseases, such as periodontitis, rheumatoid arthritis, and multiple myeloma (Zhou et al., 2019). These pro-inflammatory cytokines indirectly mediate osteoclastogenesis via a series of ways, such as increasing RANKL and M-SCF expression, reducing OPG expression, and cross-linking with the RANKL/RANK downstream pathways (Zupan et al., 2013; Rao et al., 2018). Previous studies have confirmed that Pristimerin could decrease the expression levels of pro-inflammatory cytokines in several animal models of tissue damage, such as LPS-induced acute lung injury (Shaaban et al., 2018), ovalbumin-induced allergic airway inflammation (Jin et al., 2016), and adjuvant-induced arthritis (Tong et al., 2014; Deng et al., 2015). In our study, Pristimerin reduced the expression levels of serum inflammatory factors in ovariectomized mice, including TNF-α, IL-1β, IL-6 and RANKL, which is consistent with other studies.

The limitation of our study is, although we have explored the effect of Pristimerin both on osteoclastogenesis and osteogenesis, but we didn'*t* test the effects of Pristimerin on adipogenesis. As previous studies showed, factors promoting osteogenesis are recognized as inhibitors for adipogenesis and vice versa (Kawai and Rosen, 2010). Thus elevated adipogenesis may impair osteogenesis and bone mass, experiments may take to further elucidate adipose formation after Pristimerin exposure in the next study. In addition, our experiment did not test the toxic effect of Pristimerin on other organs in mice like liver and kidney, which will be needed for the translation of Pristimerin into clinical use, much more work should be done in the future.

Our study indicated that Pristimerin has an obvious inhibitory effect on RANKL-induced osteoclastogenesis and a protective role on ovariectomy-induced bone loss. We believe our study provides persuasive evidence for developing a novel reagent to treat osteoporosis for Pristimerin in the future.

## Data Availability

The raw data supporting the conclusions of this article will be made available by the authors, without undue reservation.

## References

[B1] Adamopoulos I. E. (2018). Inflammation in bone physiology and pathology. Curr. Opin. Rheumatol. 30 (1), 59–64. 10.1097/BOR.0000000000000449 29016371 PMC5963529

[B2] Agidigbi T. S Kim C. (2019). Reactive oxygen species in osteoclast differentiation and possible pharmaceutical targets of ROS-mediated osteoclast diseases. Int. J. Mol. Sci. 20 (14). 10.3390/ijms20143576 PMC6678498 31336616

[B3] Baud'huin M. Lamoureux F. Duplomb L. Rédini F. Heymann D. (2007). RANKL, RANK, osteoprotegerin: key partners of osteoimmunology and vascular diseases. Cell. Mol. Life Sci. 64 (18), 2334–2350. 10.1007/s00018-007-7104-0 17530461 PMC11149428

[B4] Baum R. Gravallese E. M. (2016). Bone as a target organ in rheumatic disease: impact on osteoclasts and osteoblasts. Clin. Rev. Allergy Immunol. 51 (1), 1 10.1007/s12016-015-8515-6 26411424 PMC4809775

[B5] Bellezza I. Giambanco I. Minelli A. Donato R. (2018). Nrf2-Keap1 signaling in oxidative and reductive stress. Biochim. Biophys. Acta Mol. Cell Res. 1865 (5), 721–733. 10.1016/j.bbamcr.2018.02.010 29499228

[B6] Boyce B. F. (2013). Advances in the regulation of osteoclasts and osteoclast functions. J. Dent. Res. 92 (10), 860–867. 10.1177/0022034513500306 23906603 PMC3775372

[B7] Boyle W. J. Simonet W. S. Lacey D. L. (2003). Osteoclast differentiation and activation. Nature. 423 (6937), 337–342. 10.1038/nature01658 12748652

[B8] Charles J. F. Aliprantis A. O. (2014). Osteoclasts: more than ‘bone eaters'. Trends Mol. Med. 20 (8), 449–459. 10.1016/j.molmed.2014.06.001 25008556 PMC4119859

[B9] Coury F. Peyruchaud O. Machuca-Gayet I. (2019). Osteoimmunology of bone loss in inflammatory rheumatic diseases. Front. Immunol. 10, 679 10.3389/fimmu.2019.00679 31001277 PMC6456657

[B10] D'Autréaux B. Toledano M. B. (2007). ROS as signalling molecules: mechanisms that generate specificity in ROS homeostasis. Nat. Rev. Mol. Cell Biol. 8 (10), 813–824. 10.1038/nrm2256 17848967

[B11] Deeb D. Gao X. Liu Y. B. Pindolia K. Gautam S. C. (2014). Pristimerin, a quinonemethide triterpenoid, induces apoptosis in pancreatic cancer cells through the inhibition of pro-survival Akt/NF-κB/mTOR signaling proteins and anti-apoptotic Bcl-2. Int. J. Oncol. 44 (5), 1707–1715. 10.3892/ijo.2014.2325 24603988 PMC4027926

[B12] Deng Q. Bai S. Gao W. Tong L. (2015). Pristimerin inhibits angiogenesis in adjuvant-induced arthritic rats by suppressing VEGFR2 signaling pathways. Int. Immunopharm. 29 (2), 302–313. 10.1016/j.intimp.2015.11.001 26548348

[B13] Dos Santos V. A. Dos Santos D. P. Castro-Gamboa I. Zanoni M. V. Furlan M. (2010). Evaluation of antioxidant capacity and synergistic associations of quinonemethide triterpenes and phenolic substances from Maytenus ilicifolia (Celastraceae). Molecules. 15 (10), 6956–6973. 10.3390/molecules15106956 20938406 PMC6259563

[B14] El-Agamy D. S. Shaaban A. A. Almaramhy H. H. Elkablawy S. Elkablawy M. A. (2018). Pristimerin as a novel hepatoprotective agent against experimental autoimmune hepatitis. Front. Pharmacol. 9, 292 10.3389/fphar.2018.00292 29643811 PMC5883828

[B15] El-Agamy D. S. El-Harbi K. M. Khoshhal S. Ahmed N. Elkablawy M. A. Shaaban A. A. (2019). Pristimerin protects against doxorubicin-induced cardiotoxicity and fibrosis through modulation of Nrf2 and MAPK/NF-kB signaling pathways. Canc. Manag. Res. 11, 47–61. 10.2147/CMAR.S186696 PMC6304079 30588110

[B16] Ensrud K. E. Crandall C. J. (2017). Osteoporosis. Ann. Intern. Med. 167 (3), ITC17–ITC32. 10.7326/AITC201708010 28761958

[B17] Feng X. McDonald J. M. (2011). Disorders of bone remodeling. Annu. Rev. Pathol. 6, 121–145. 10.1146/annurev-pathol-011110-130203 20936937 PMC3571087

[B18] Figueiredo J. N. Räz B. Séquin U. (1998). Novel quinone methides from Salacia kraussii with *in vitro* antimalarial activity. J. Nat. Prod. 61 (6), 718–723. 10.1021/np9704157 9644053

[B19] Guan H. Zhao L. Cao H. Chen A. Xiao J. (2015). Epoxyeicosanoids suppress osteoclastogenesis and prevent ovariectomy-induced bone loss. Faseb. J. 29 (3), 1092–1101. 10.1096/fj.14-262055 25466887

[B20] Gullo F. P. Sardi J. C. Santos V. A. Sangalli-Leite F. Pitangui N. S. Rossi S. A. (2012). Antifungal activity of maytenin and pristimerin. Evid Based Complement Alternat Med. 2012, 340787 10.1155/2012/340787 22675379 PMC3364566

[B21] Hui B. Yao X. Zhou Q. Wu Z. Sheng P. Zhang L. (2014). Pristimerin, a natural anti-tumor triterpenoid, inhibits LPS-induced TNF-α and IL-8 production through down-regulation of ROS-related classical NF-κB pathway in THP-1 cells. Int. Immunopharm. 21 (2), 501–508. 10.1016/j.intimp.2014.06.010 24957686

[B22] Hui B. Zhang L. Zhou Q. Hui L. (2018). Pristimerin inhibits LPS-triggered neurotoxicity in BV-2 microglia cells through modulating IRAK1/TRAF6/TAK1-mediated NF-κB and AP-1 signaling pathways in vitro. Neurotox. Res. 33 (2), 268–283. 10.1007/s12640-017-9837-3 29119451

[B23] Jin Y. Wang Y. Zhao D. Ma S. Lu J. Shuang G. (2016). Pristimerin attenuates ovalbumin-induced allergic airway inflammation in mice. Immunopharmacol. Immunotoxicol. 38 (3), 221–227. 10.3109/08923973.2016.1168435 27098091

[B24] Kawai M. Rosen C. J. (2010). PPARγ: a circadian transcription factor in adipogenesis and osteogenesis. Nat. Rev. Endocrinol. 6 (11), 629–636. 10.1038/nrendo.2010.155 20820194 PMC3132113

[B25] Kim H. J. Park G. M. Kim J. K. (2013). Anti-inflammatory effect of pristimerin on lipopolysaccharide-induced inflammatory responses in murine macrophages. Arch Pharm. Res. (Seoul). 36 (4), 495–500. 10.1007/s12272-013-0054-1 23435916

[B26] Li J. J. Yan Y. Y. Sun H. M. Liu Y. Su C. Y. Chen H. B. (2019). Anti-Cancer effects of pristimerin and the mechanisms: a critical review. Front. Pharmacol. 10, 746 10.3389/fphar.2019.00746 31354475 PMC6640652

[B27] Liu H. Dong Y. Gao Y. Zhao L. Cai C. Qi D. (2019). Hesperetin suppresses RANKL-induced osteoclastogenesis and ameliorates lipopolysaccharide-induced bone loss. J. Cell. Physiol. 234 (7), 11009–11022. 10.1002/jcp.27924 30548260

[B28] Luo D. Q. Wang H. Tian X. Shao H. J. Liu J. K. (2005). Antifungal properties of pristimerin and celastrol isolated from Celastrus hypoleucus. Pest Manag. Sci. 61 (1), 85–90. 10.1002/ps.953 15593077

[B29] Mittal M. Siddiqui M. R. Tran K. Reddy S. P. Malik A. B. (2014). Reactive oxygen species in inflammation and tissue injury. Antioxidants Redox Signal. 20 (7), 1126–1167. 10.1089/ars.2012.5149 PMC3929010 23991888

[B30] Mundy G. R. (2007). Osteoporosis and inflammation. Nutr. Rev. 65 (12), 147–151. 10.1301/nr.2007.dec.S147-S151 18240539

[B31] Nakashima T. Takayanagi H. (2011). New regulation mechanisms of osteoclast differentiation. Ann. N. Y. Acad. Sci. 1240, E13–E18. 10.1111/j.1749-6632.2011.06373.x 22360322

[B32] Ono T. Nakashima T. (2018). Recent advances in osteoclast biology. Histochem. Cell Biol. 149 (4), 325–341. 10.1007/s00418-018-1636-2 29392395

[B33] Prasad S. Gupta S. C. Tyagi A. K. (2017). Reactive oxygen species (ROS) and cancer: role of antioxidative nutraceuticals. Canc. Lett. 387, 95 10.1016/j.canlet.2016.03.042 27037062

[B34] Rao S. S. Hu Y. Xie P. L. Cao J. Wang Z. X. Liu J. H. (2018). Omentin-1 prevents inflammation-induced osteoporosis by downregulating the pro-inflammatory cytokines. Bone Res. 6, 9 10.1038/s41413-018-0012-0 29619269 PMC5876344

[B35] Roodman G. D. Windle J. J. (2005). Paget disease of bone. J. Clin. Invest. 115 (2), 200–208. 10.1172/JCI24281 15690073 PMC546434

[B36] Shaaban A. A. El-Kashef D. H. Hamed M. F. El-Agamy D. S. (2018). Protective effect of pristimerin against LPS-induced acute lung injury in mice. Int. Immunopharm. 59, 31–39. 10.1016/j.intimp.2018.03.033 29621734

[B37] Siddiqui J. A. Partridge N. C. (2016). Physiological bone remodeling: systemic regulation and growth factor involvement. Physiology. 31 (3), 233–245. 10.1152/physiol.00061.2014 27053737 PMC6734079

[B38] Sun Y. X. Xu A. H. Yang Y. Li J. (2015). Role of Nrf2 in bone metabolism. J. Biomed. Sci. 22, 101 10.1186/s12929-015-0212-5 26511009 PMC4625735

[B39] Sun X. Xie Z. Hu B. Zhang B. Ma Y. Pan X. (2020). The Nrf2 activator RTA-408 attenuates osteoclastogenesis by inhibiting STING dependent NF-κb signaling. Redox Biol. 28, 101309 10.1016/j.redox.2019.101309 31487581 PMC6728880

[B40] Takayanagi H. Kim S. Koga T. Nishina H. Isshiki M. Yoshida H. (2002). Induction and activation of the transcription factor NFATc1 (NFAT2) integrate RANKL signaling in terminal differentiation of osteoclasts. Dev. Cell. 3 (6), 889–901. 10.1016/s1534-5807(02)00369-6 12479813

[B41] Tonelli C. Chio I. I. C. Tuveson D. A. (2018). Transcriptional regulation by Nrf2. Antioxidants Redox Signal. 29 (17), 1727–1745. 10.1089/ars.2017.7342 PMC6208165 28899199

[B42] Tong L. Nanjundaiah S. M. Venkatesha S. H. Astry B. Yu H. Moudgil K. D. (2014). Pristimerin, a naturally occurring triterpenoid, protects against autoimmune arthritis by modulating the cellular and soluble immune mediators of inflammation and tissue damage. Clin. Immunol. 155 (2), 220–230. 10.1016/j.clim.2014.09.014 25308129 PMC8136150

[B43] Tu Y. Tan F. Zhou J. Pan J. (2018). Pristimerin targeting NF-κB pathway inhibits proliferation, migration, and invasion in esophageal squamous cell carcinoma cells. Cell Biochem. Funct. 36 (4), 228–240. 10.1002/cbf.3335 29781107

[B44] Wada T. Nakashima T. Hiroshi N. Penninger J. M. (2006). RANKL-RANK signaling in osteoclastogenesis and bone disease. Trends Mol. Med. 12 (1), 17–25. 10.1016/j.molmed.2005.11.007 16356770

[B45] Walsh M. C. Choi Y. (2014). Biology of the RANKL-RANK-OPG system in immunity, bone, and beyond. Front. Immunol. 5, 511 10.3389/fimmu.2014.00511 25368616 PMC4202272

[B46] Watanabe J. Yamada M. Niibe K. Zhang M. Kondo T. Ishibashi M. (2018). Preconditioning of bone marrow-derived mesenchymal stem cells with N-acetyl-L-cysteine enhances bone regeneration via reinforced resistance to oxidative stress. Biomaterials. 185, 25–38. 10.1016/j.biomaterials.2018.08.055 30216807

[B47] Wilson S. R. Peters C. Saftig P. Brömme D. (2009). Cathepsin K activity-dependent regulation of osteoclast actin ring formation and bone resorption. J. Biol. Chem. 284 (4), 2584–2592. 10.1074/jbc.M805280200 19028686 PMC2629117

[B48] Xie X. Xie S. Xie C. Fang Y. Li Z. Wang R. (2019). Pristimerin attenuates cell proliferation of uveal melanoma cells by inhibiting insulin-like growth factor-1 receptor and its downstream pathways. J. Cell Mol. Med. 23 (11), 7545–7553. 10.1111/jcmm.14623 31508890 PMC6815816

[B49] Yousef B. A. Hassan H. M. Zhang L. Y. Jiang Z. Z. (2018). Pristimerin exhibits *in vitro* and *in vivo* anticancer activities through inhibition of nuclear factor-κB signaling pathway in colorectal cancer cells. Phytomedicine. 40, 140–147. 10.1016/j.phymed.2018.01.008 29496166

[B50] Yadav V. R. Prasad S. Sung B. Kannappan R. Aggarwal B. B. (2010). Targeting inflammatory pathways by triterpenoids for prevention and treatment of cancer. Toxins. 2 (10), 2428–2466. 10.3390/toxins2102428 22069560 PMC3153165

[B51] Zhou M. Li S. Pathak J. L. (2019). Pro-inflammatory cytokines and osteocytes. Curr. Osteoporos. Rep. 17 (3, 97). 10.1007/s11914-019-00507-z 30915637

[B52] Zupan J. Jeras M. Marc J. (2013). Osteoimmunology and the influence of pro-inflammatory cytokines on osteoclasts. Biochem. Med. 23 (1), 43–63. 10.11613/bm.2013.007 PMC3900089 23457765

