# Supplementary figures and images for: Pristimerin Suppresses RANKL-Induced Osteoclastogenesis and Ameliorates Ovariectomy-Induced Bone Loss
Source: Front Pharmacol. 2021 Jan 15;11:621110. doi: 10.3389/fphar.2020.621110 (PMC7898668; doi:10.3389/fphar.2020.621110)

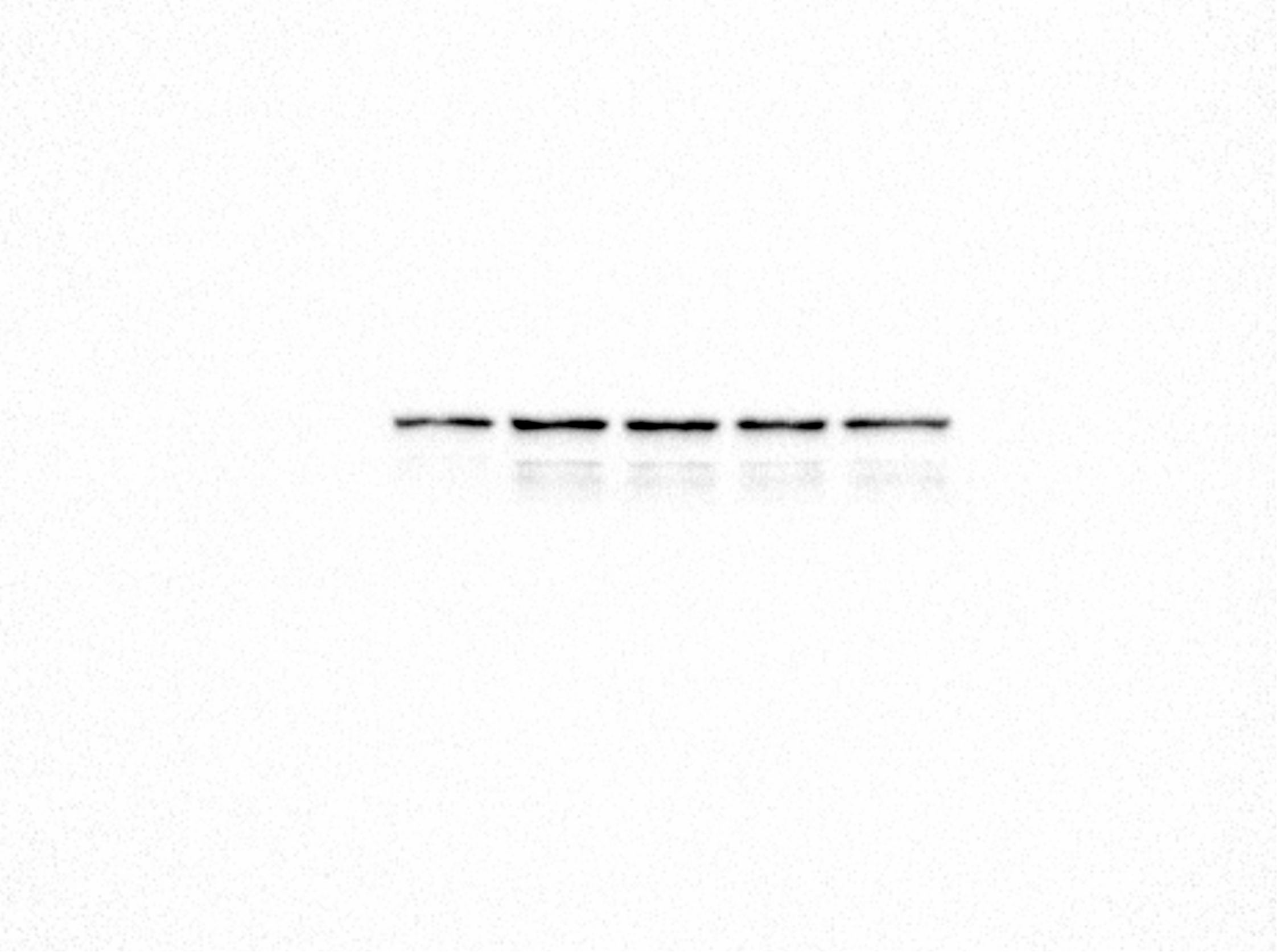

Supplement: Supplementary file 1 [file presentation1.zip › original image files/Figure 3B c-Fos.tif]

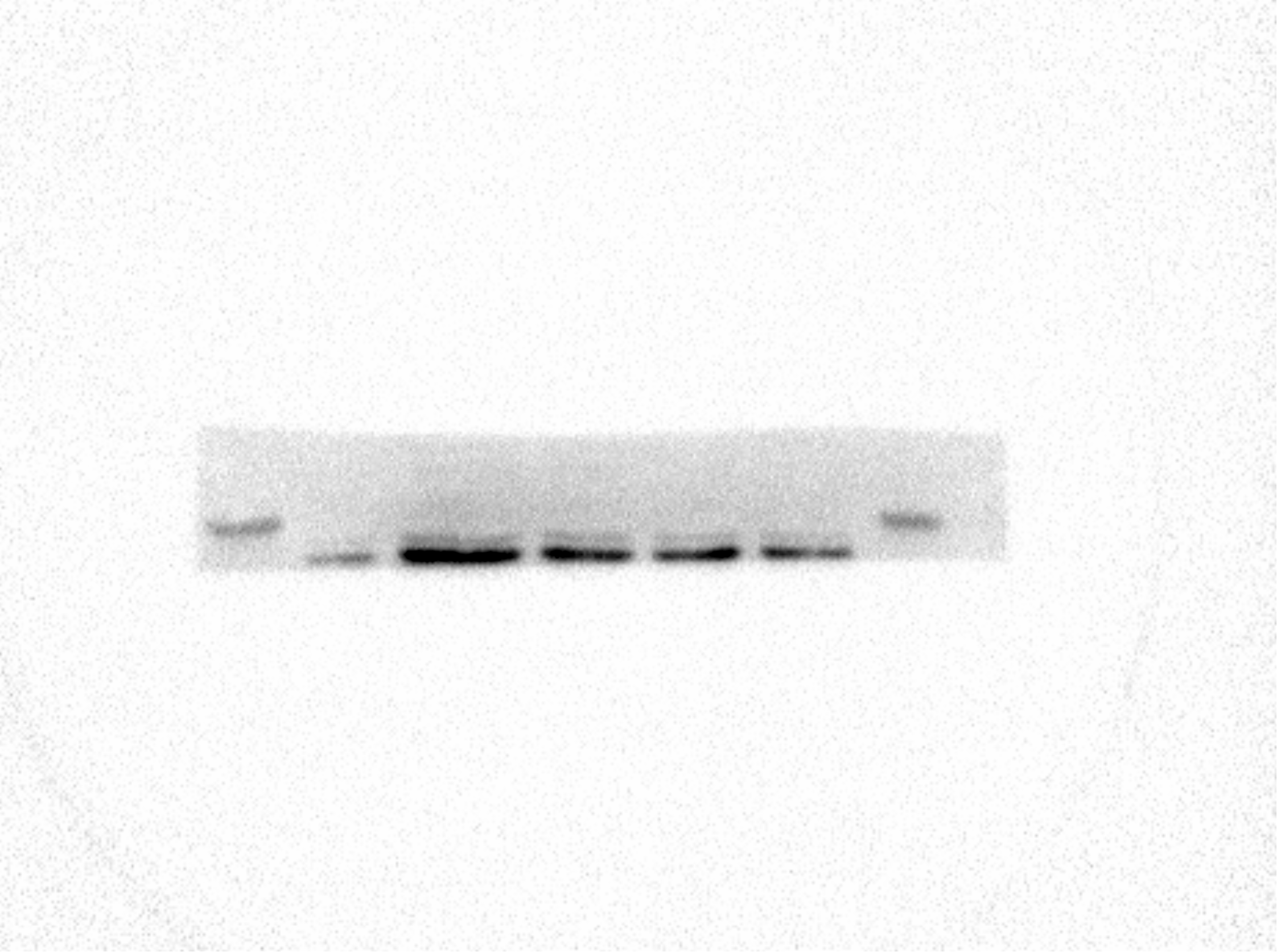

Supplement: Supplementary file 1 [file presentation1.zip › original image files/Figure 3B CTSK.tif]

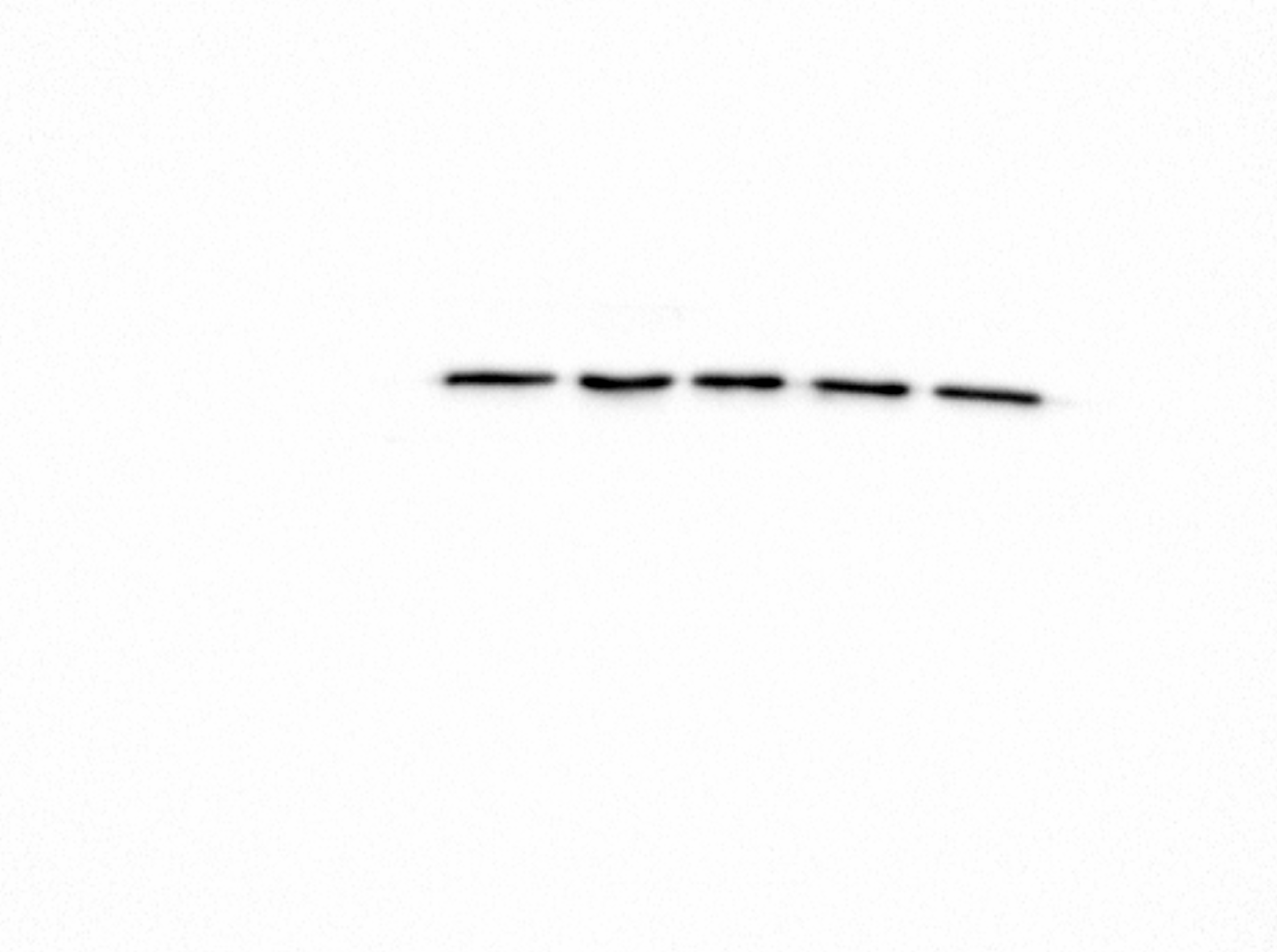

Supplement: Supplementary file 1 [file presentation1.zip › original image files/Figure 3B GAPDH.tif]

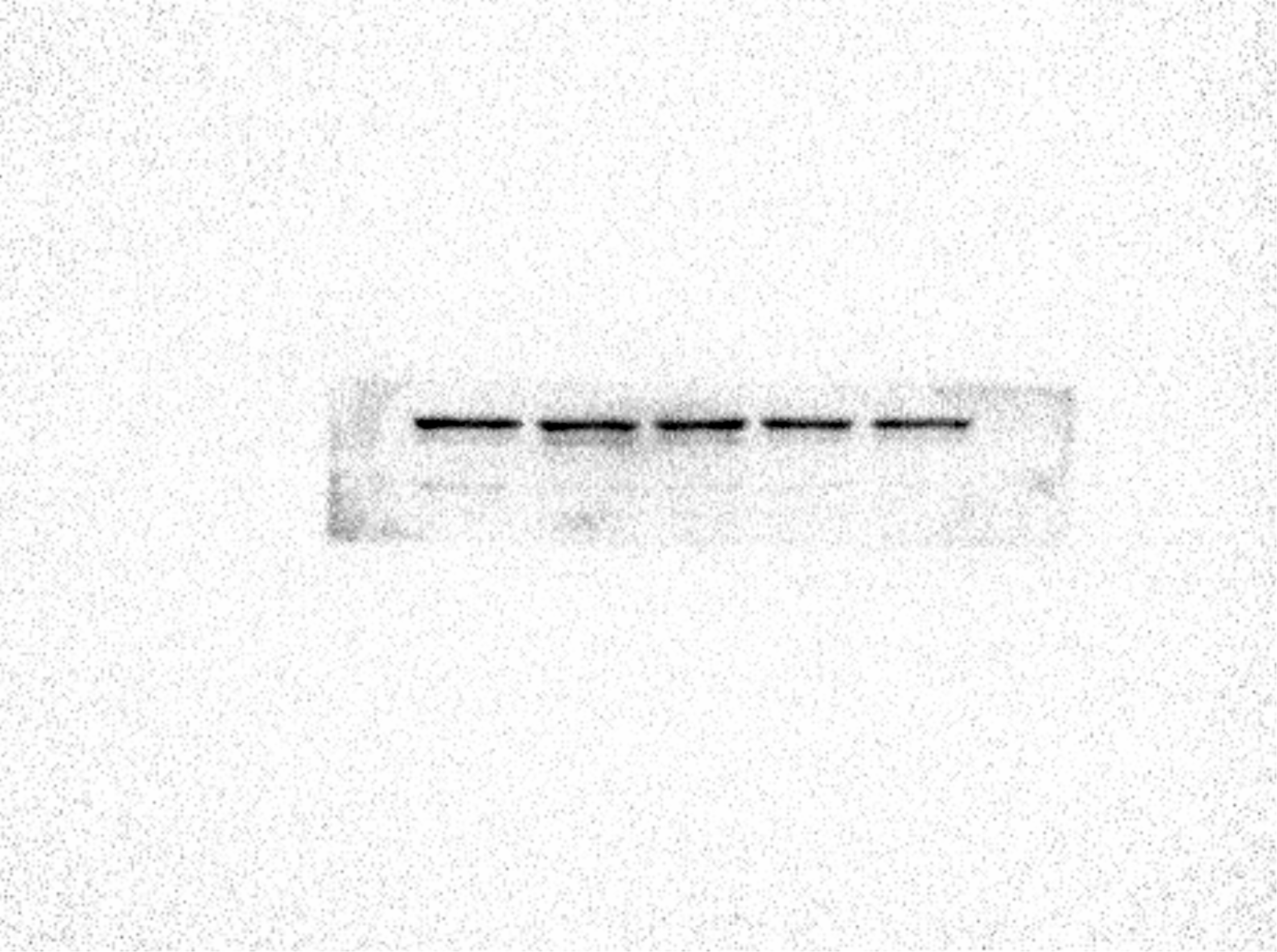

Supplement: Supplementary file 1 [file presentation1.zip › original image files/Figure 3B MMP-9.tif]

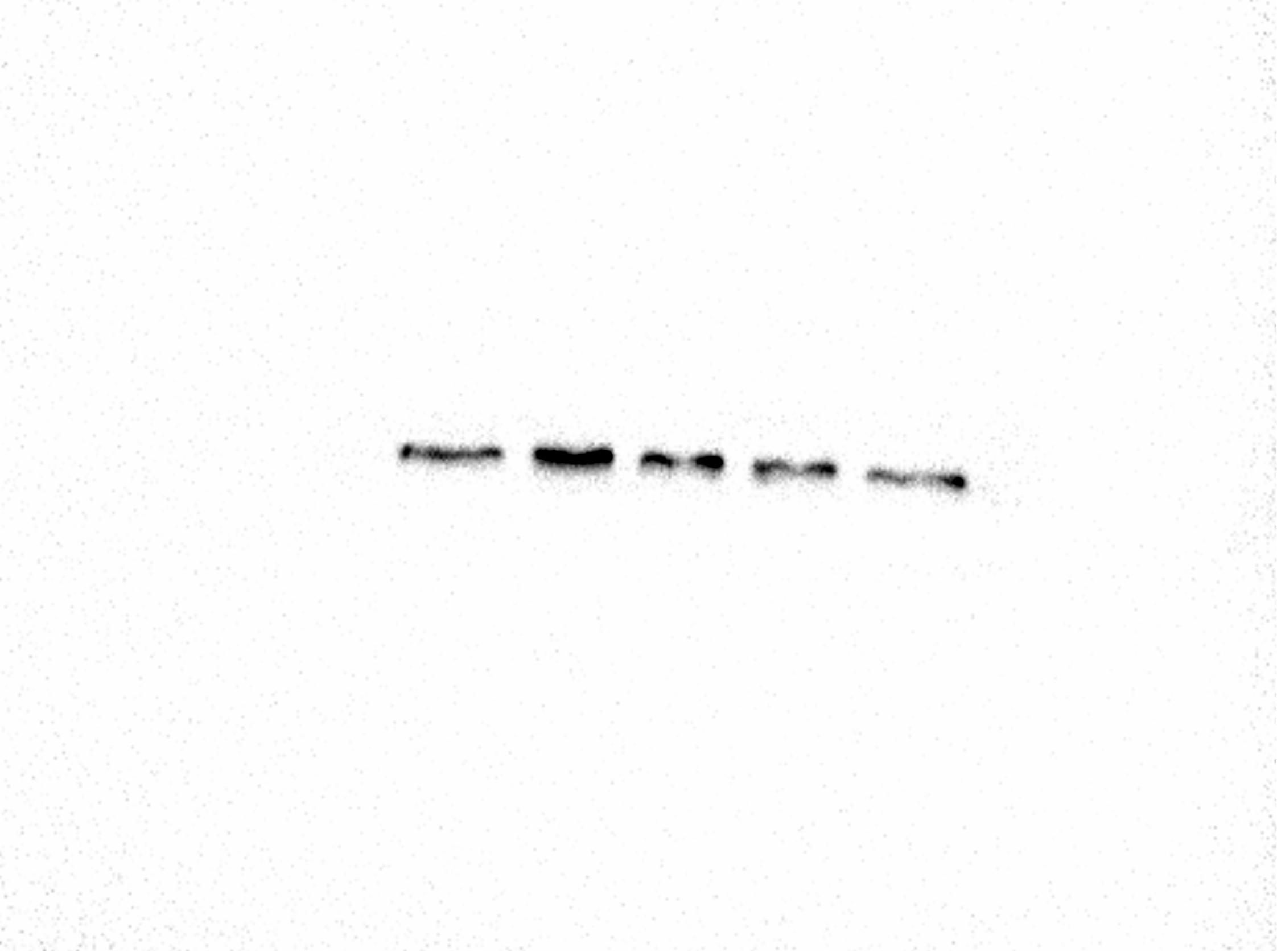

Supplement: Supplementary file 1 [file presentation1.zip › original image files/Figure 3B NFATc1.tif]

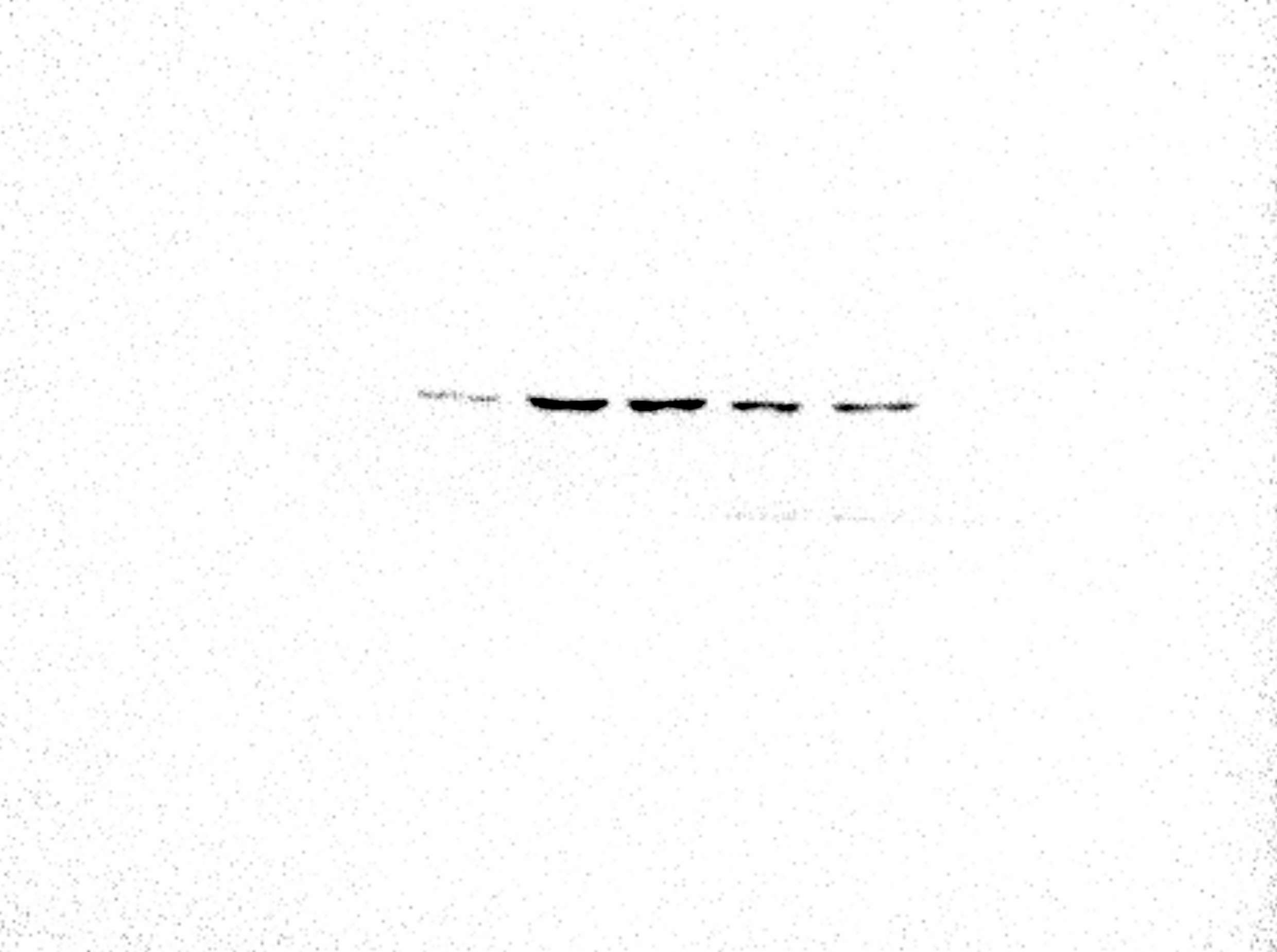

Supplement: Supplementary file 1 [file presentation1.zip › original image files/Figure 3B TRAP.tif]

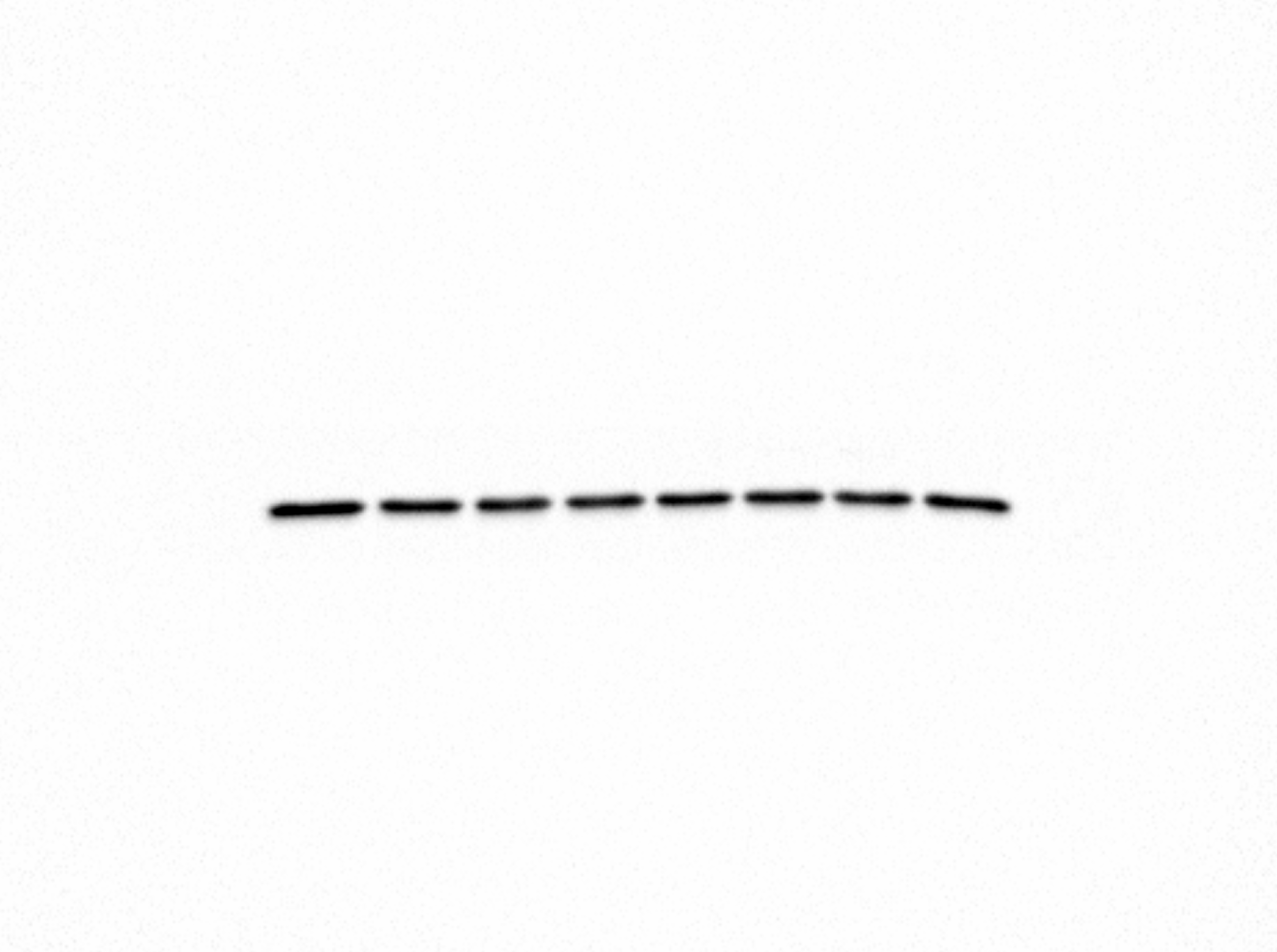

Supplement: Supplementary file 1 [file presentation1.zip › original image files/Figure 4A GAPDH.tif]

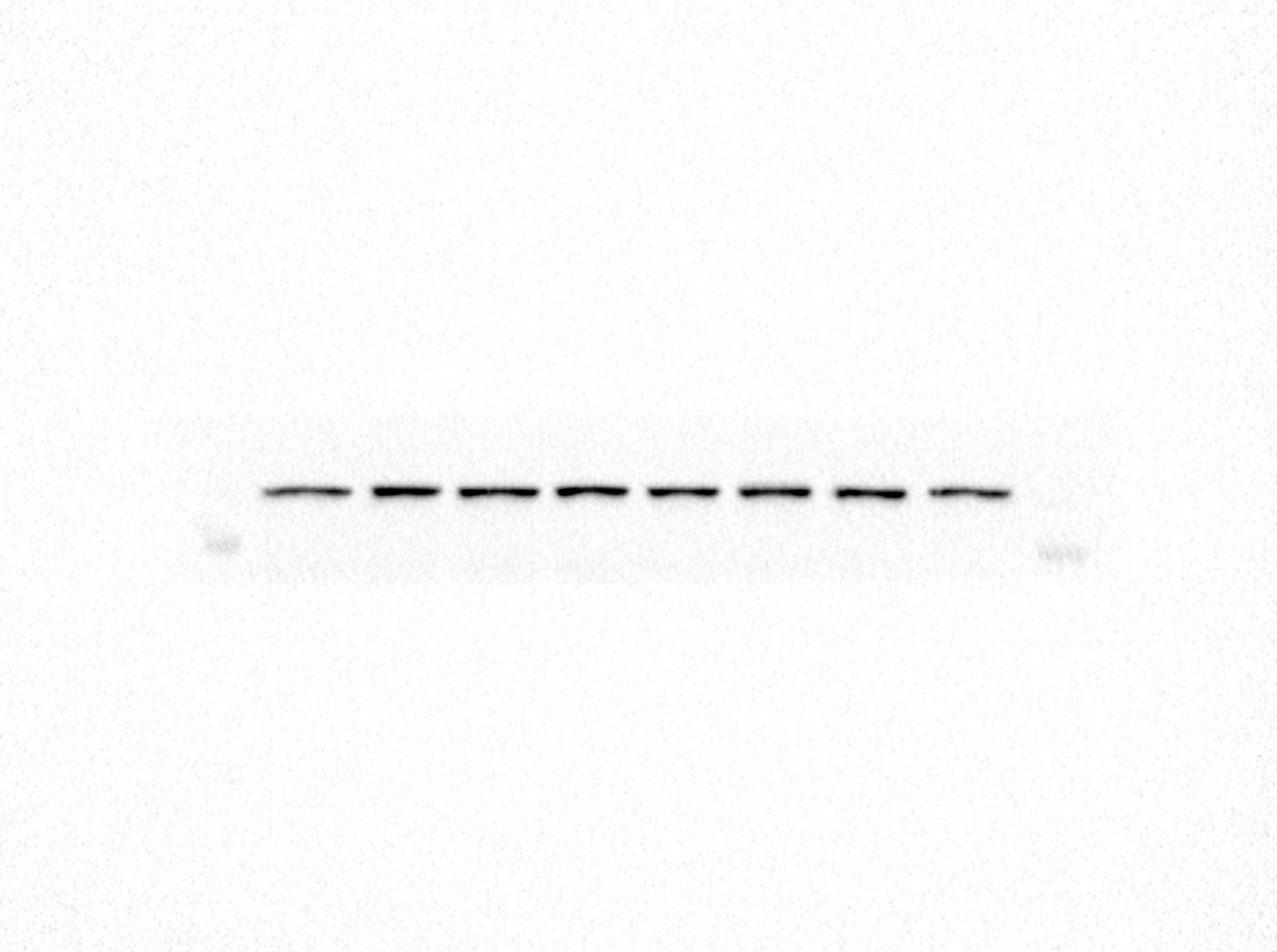

Supplement: Supplementary file 1 [file presentation1.zip › original image files/Figure 4A IKKa┬.tif]

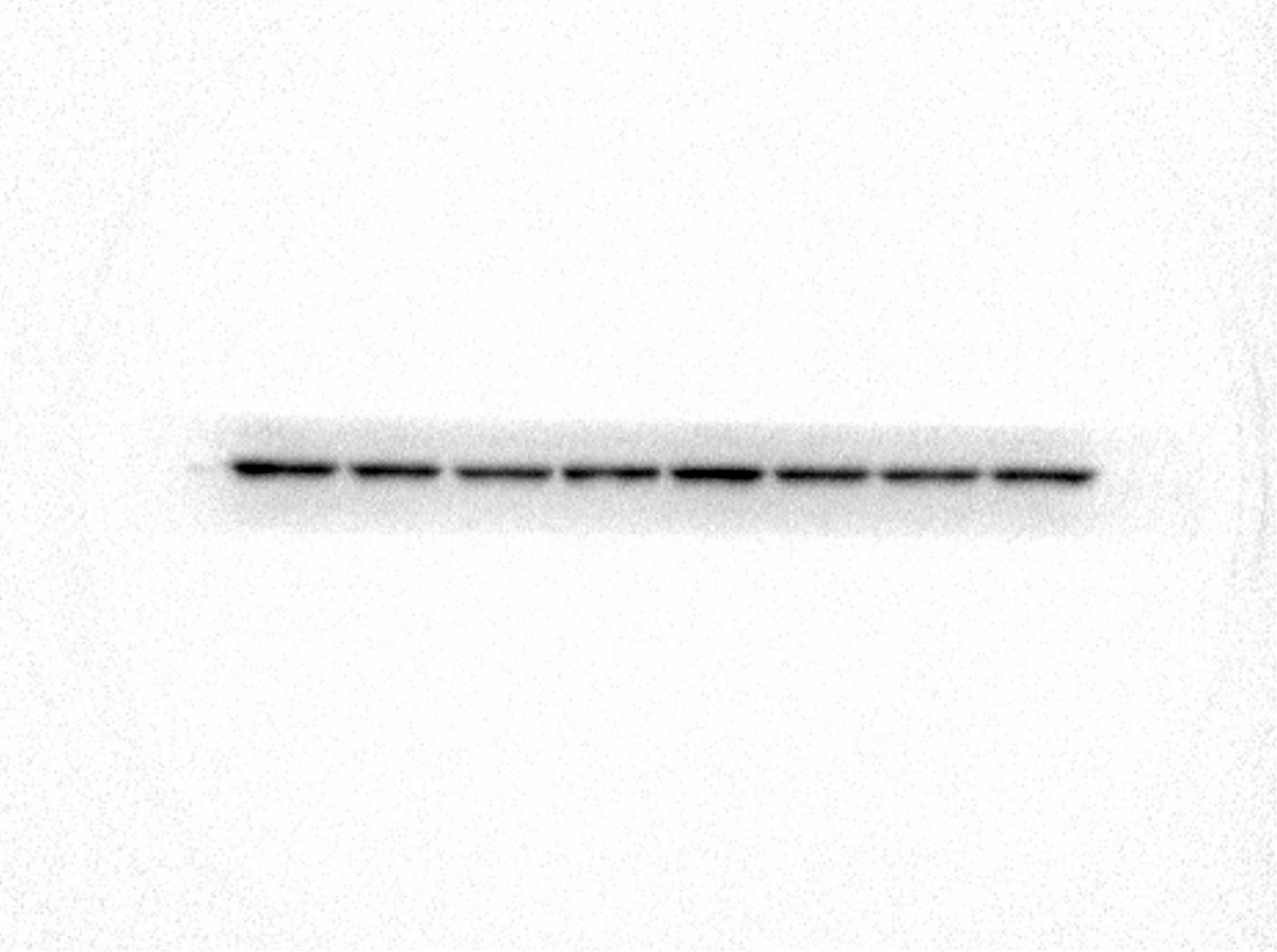

Supplement: Supplementary file 1 [file presentation1.zip › original image files/Figure 4A Ia╩Ba┴.tif]

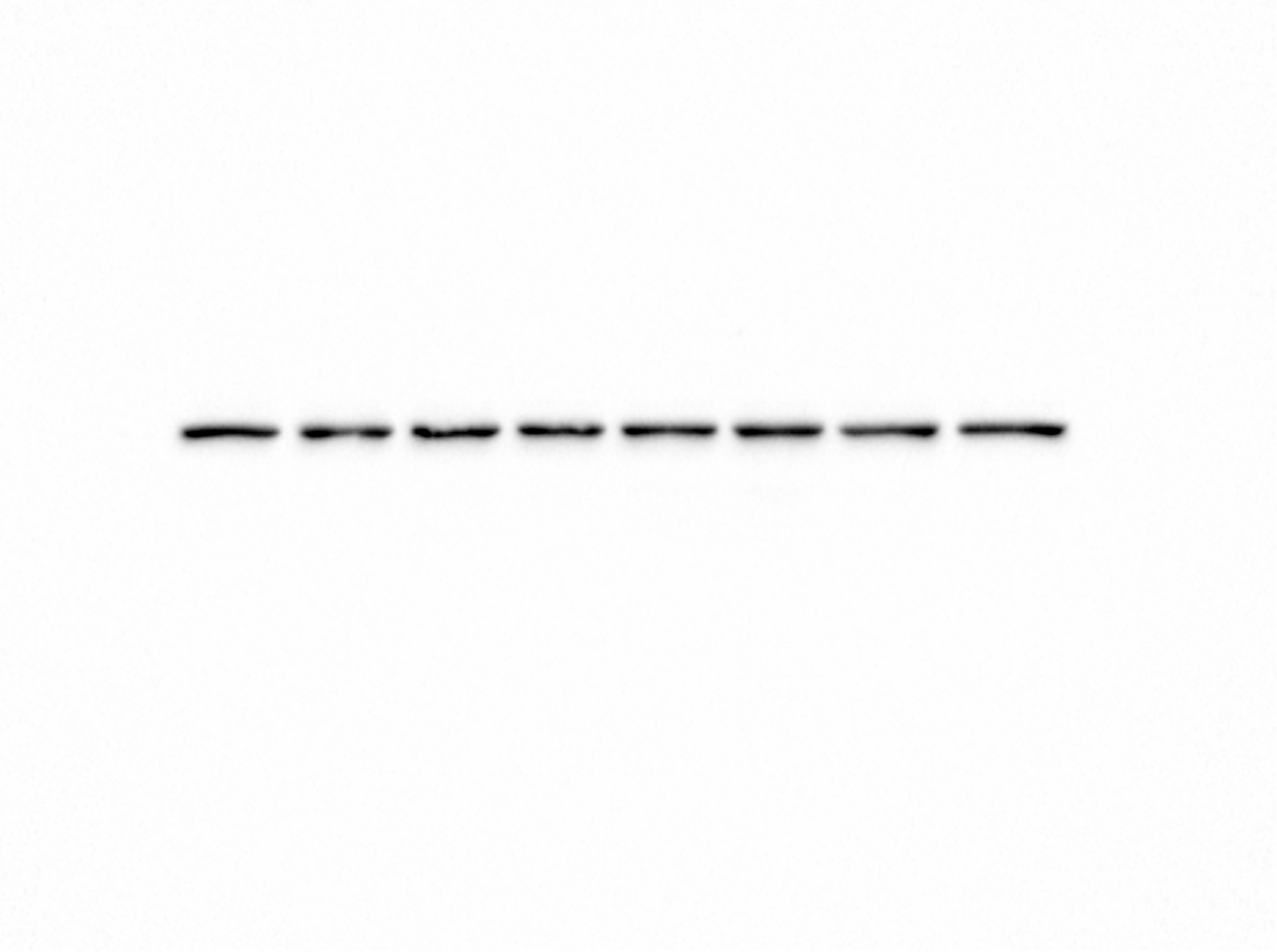

Supplement: Supplementary file 1 [file presentation1.zip › original image files/Figure 4A P65.tif]

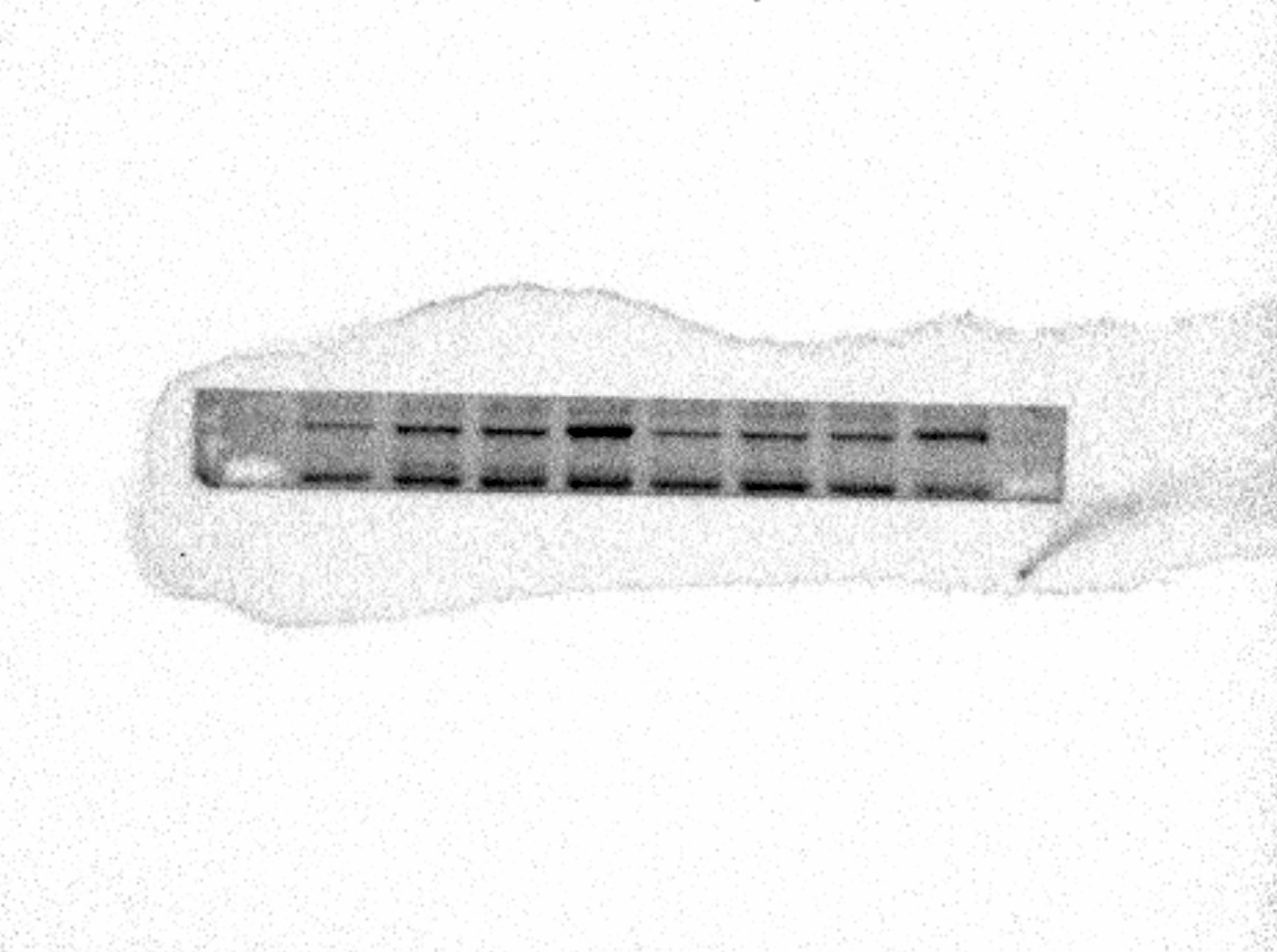

Supplement: Supplementary file 1 [file presentation1.zip › original image files/Figure 4A p-IKKa┴a┬.tif]

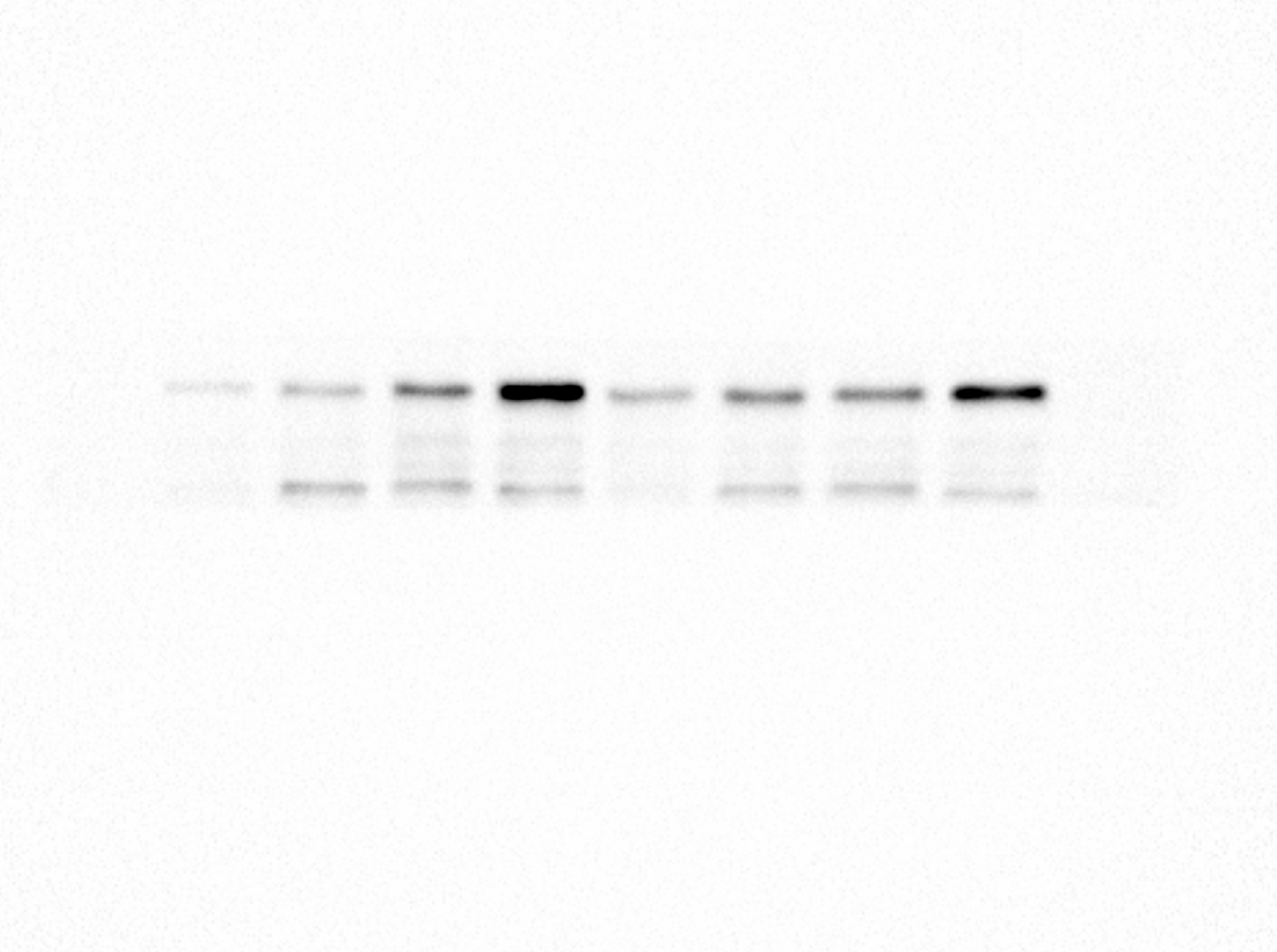

Supplement: Supplementary file 1 [file presentation1.zip › original image files/Figure 4A p-Ia╩Ba┴.tif]

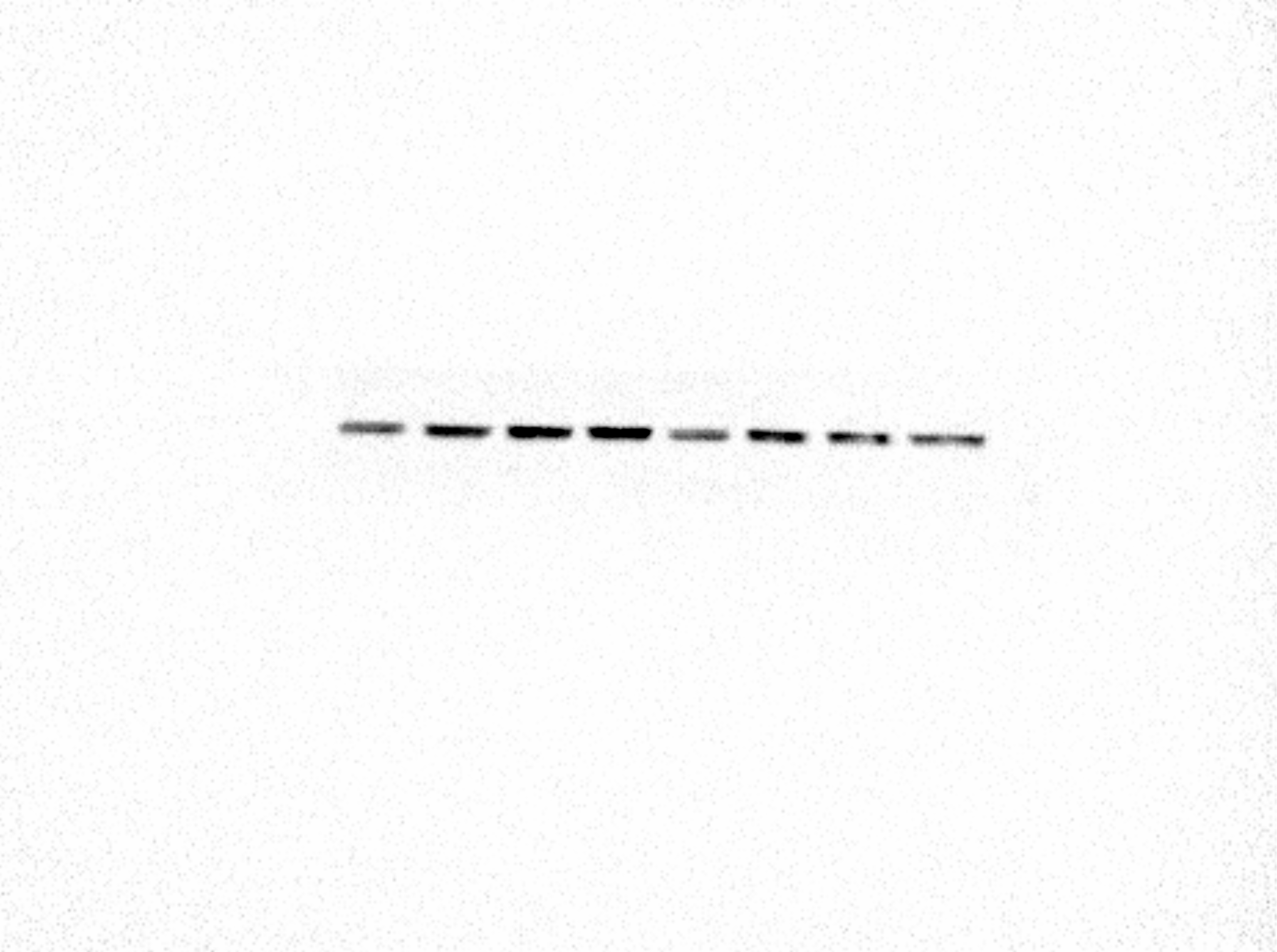

Supplement: Supplementary file 1 [file presentation1.zip › original image files/Figure 4A p-P65.tif]

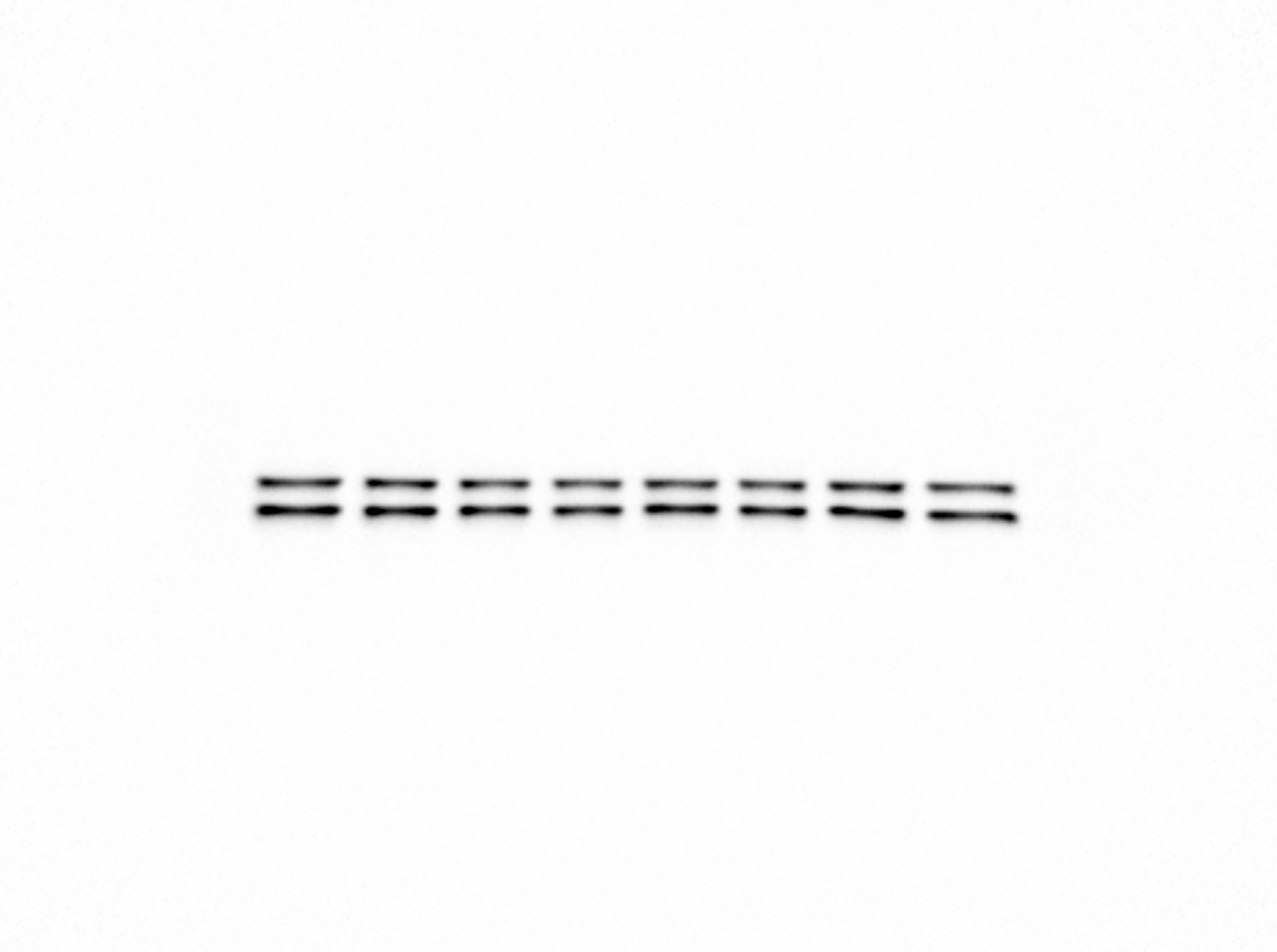

Supplement: Supplementary file 1 [file presentation1.zip › original image files/Figure 4B ERK.tif]

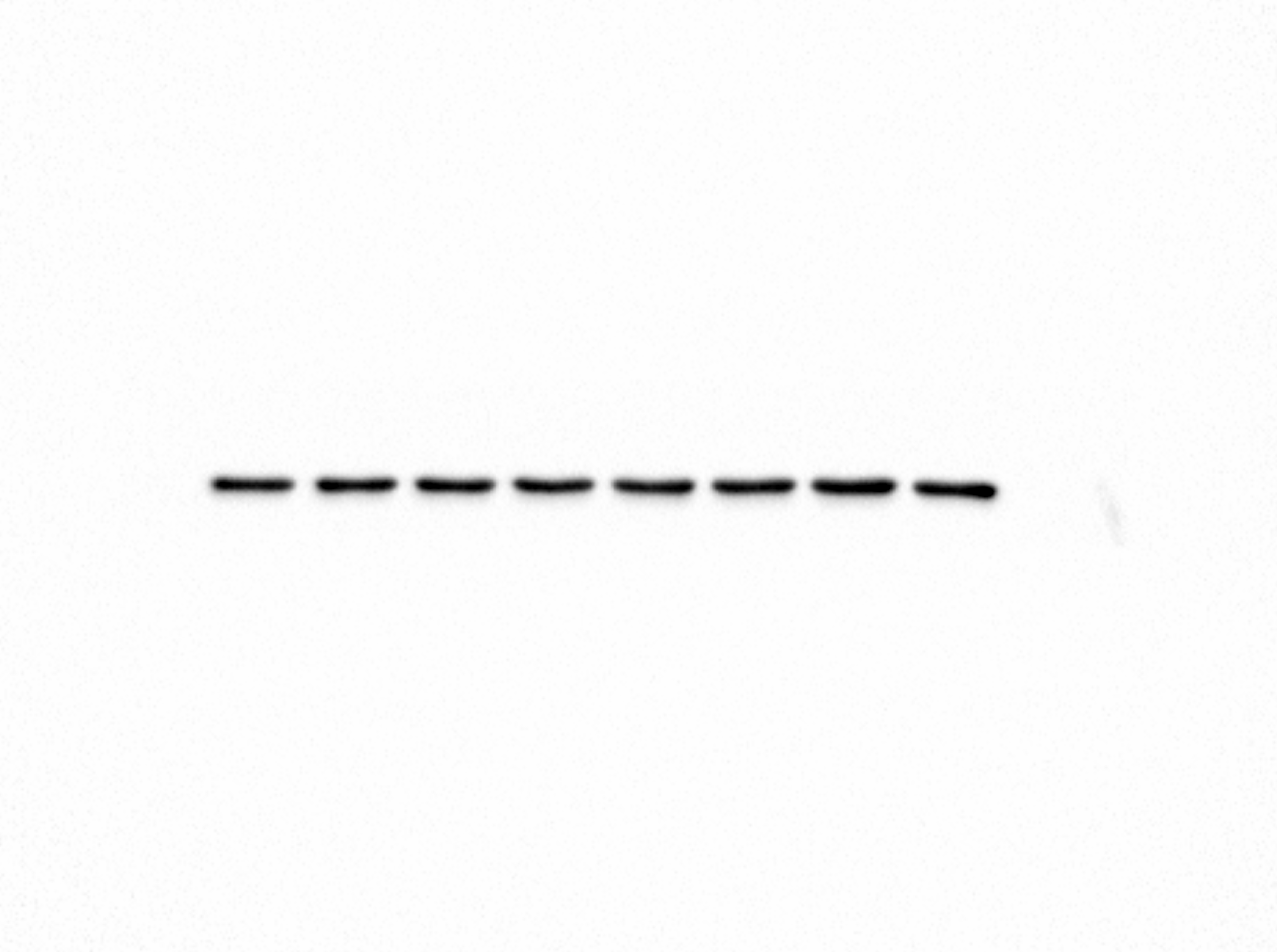

Supplement: Supplementary file 1 [file presentation1.zip › original image files/Figure 4B GAPDH.tif]

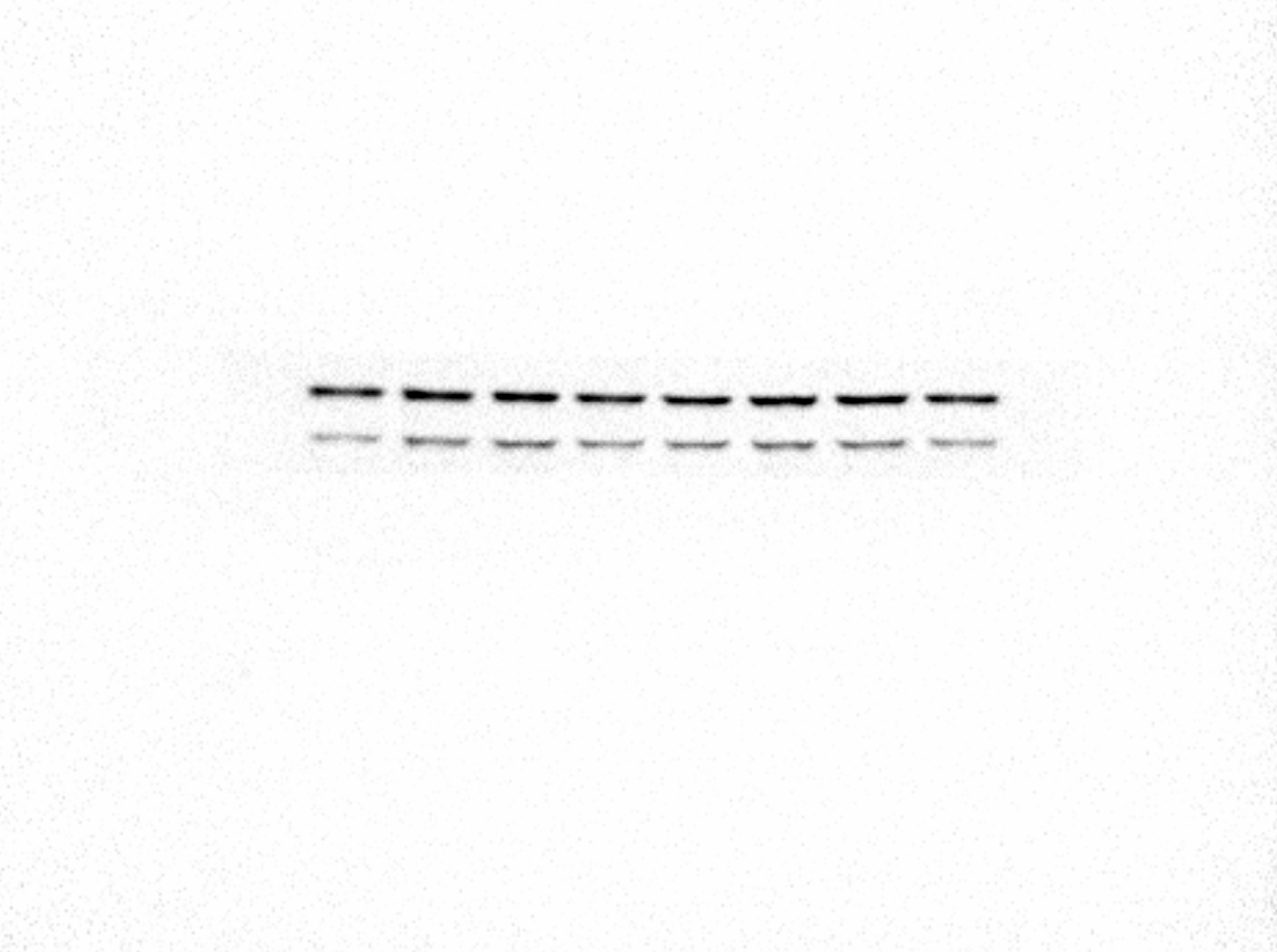

Supplement: Supplementary file 1 [file presentation1.zip › original image files/Figure 4B JNK.tif]

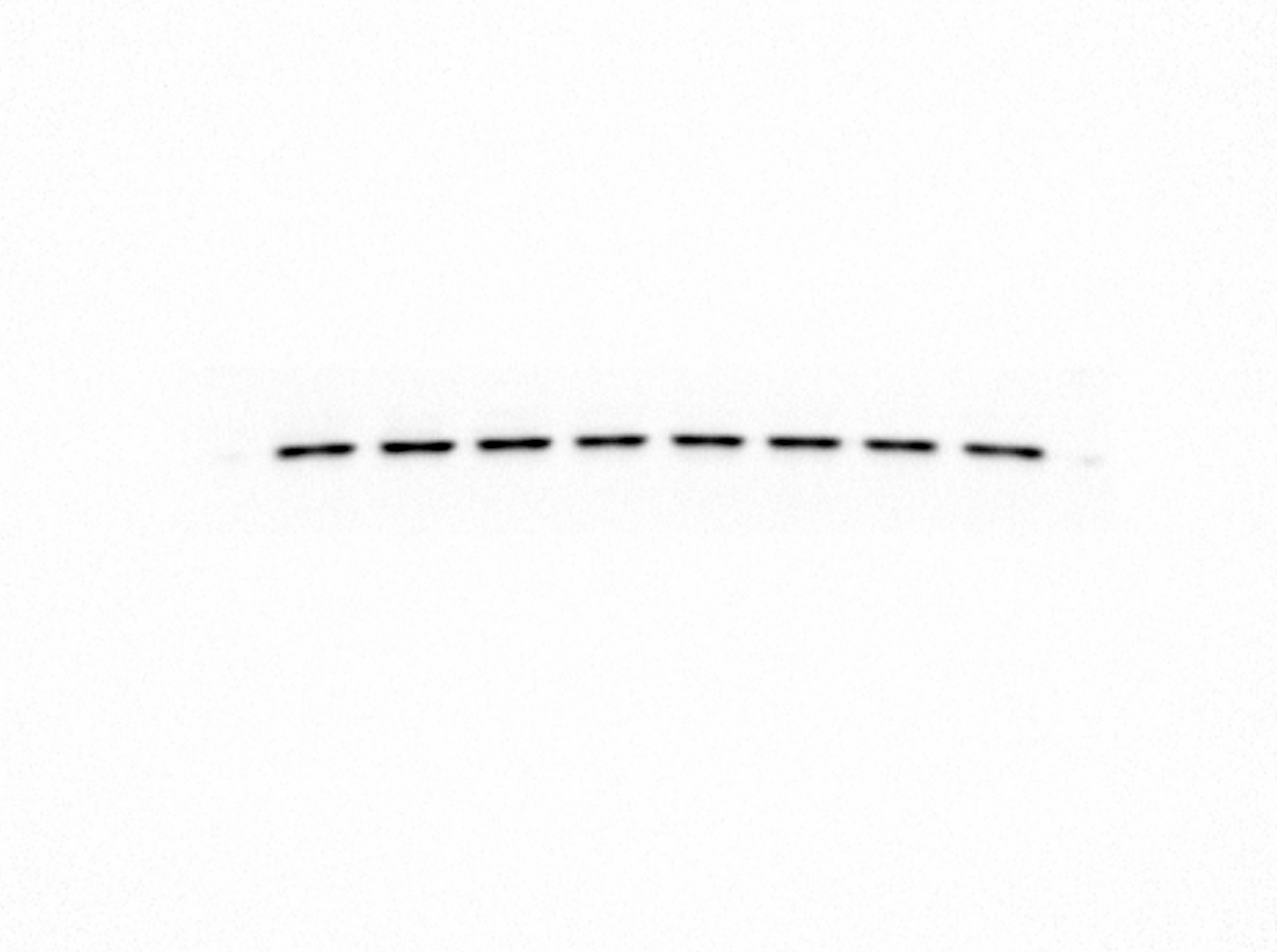

Supplement: Supplementary file 1 [file presentation1.zip › original image files/Figure 4B P38.tif]

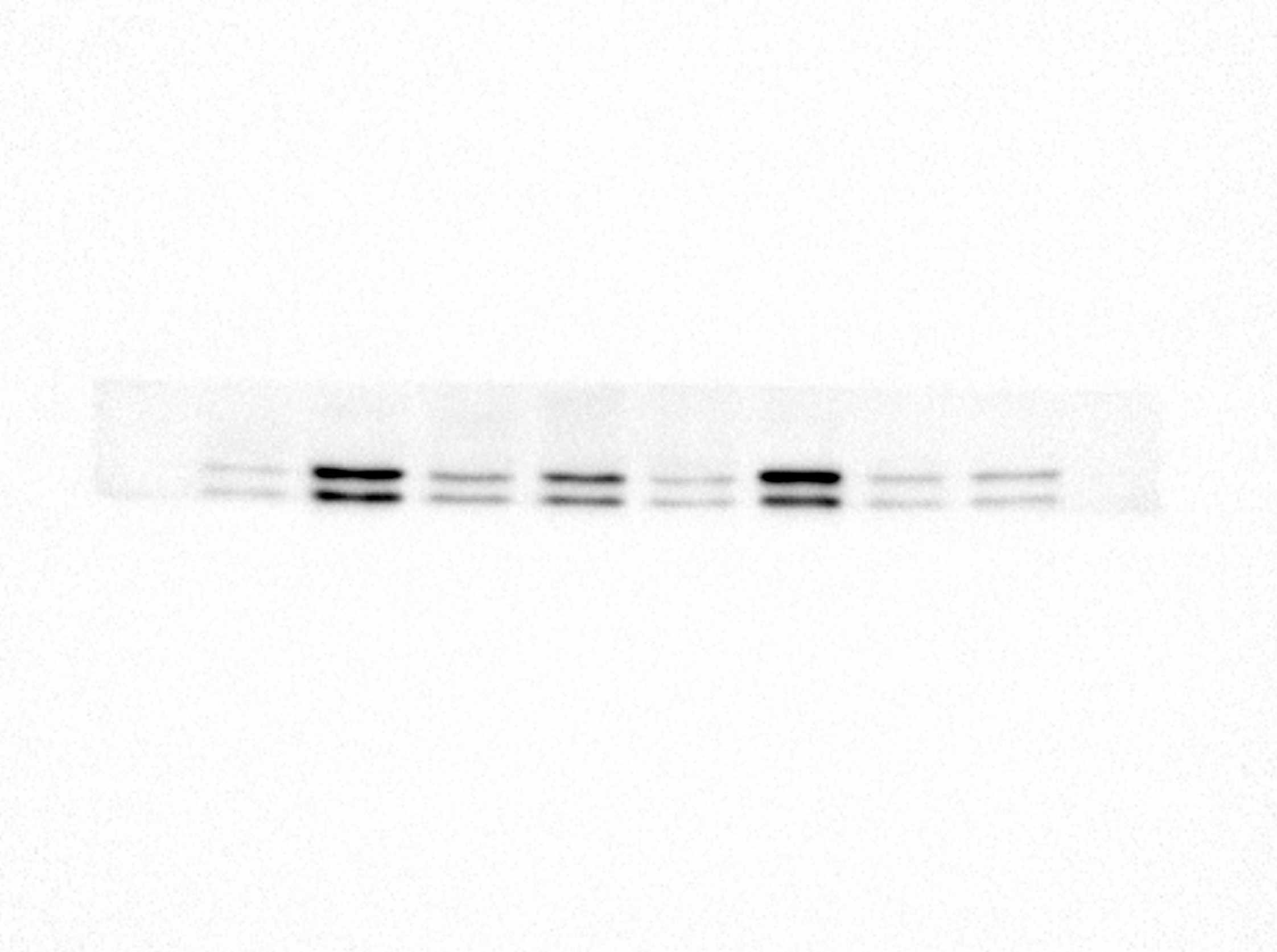

Supplement: Supplementary file 1 [file presentation1.zip › original image files/Figure 4B p-ERK.tif]

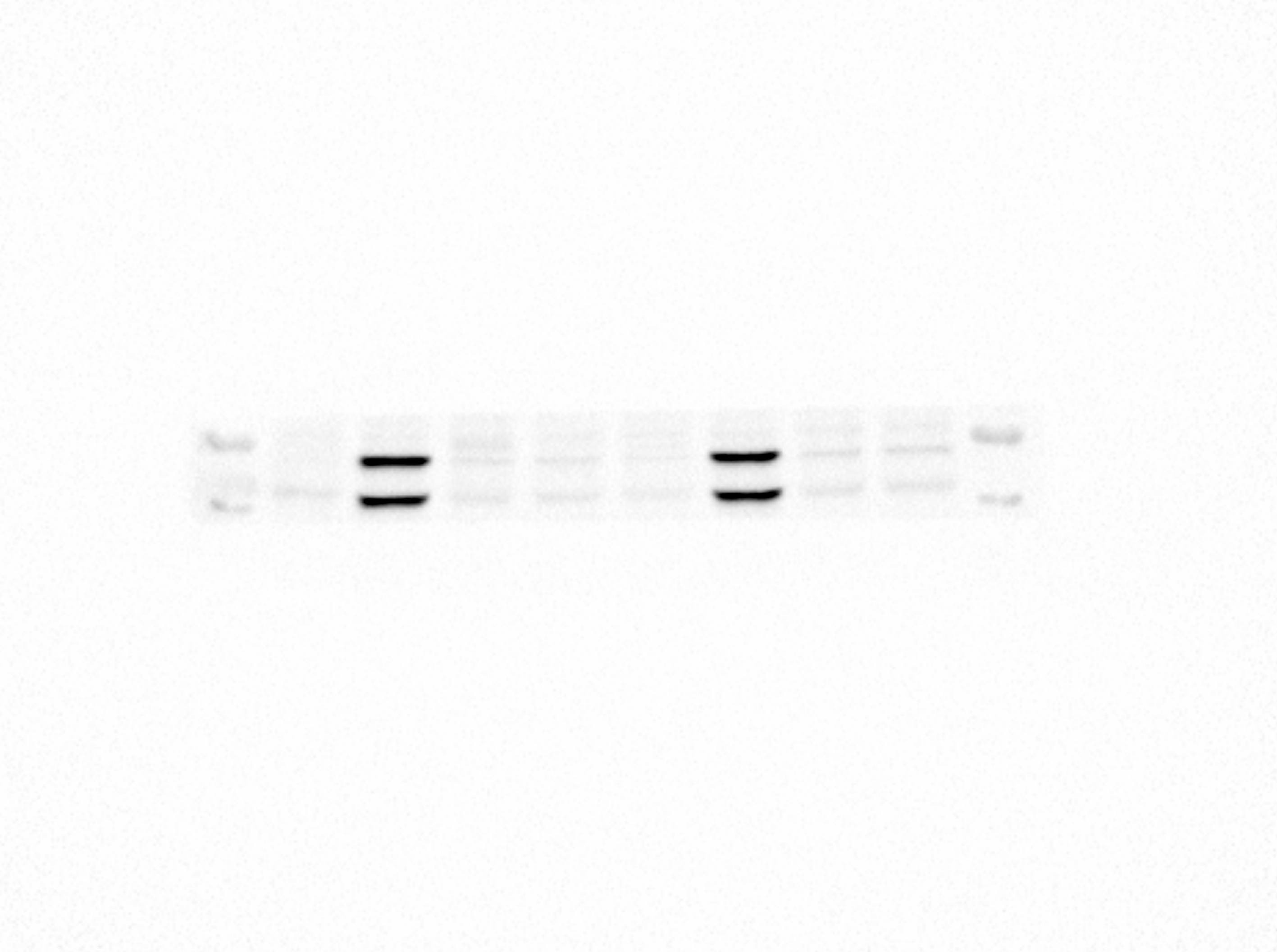

Supplement: Supplementary file 1 [file presentation1.zip › original image files/Figure 4B p-JNK.tif]

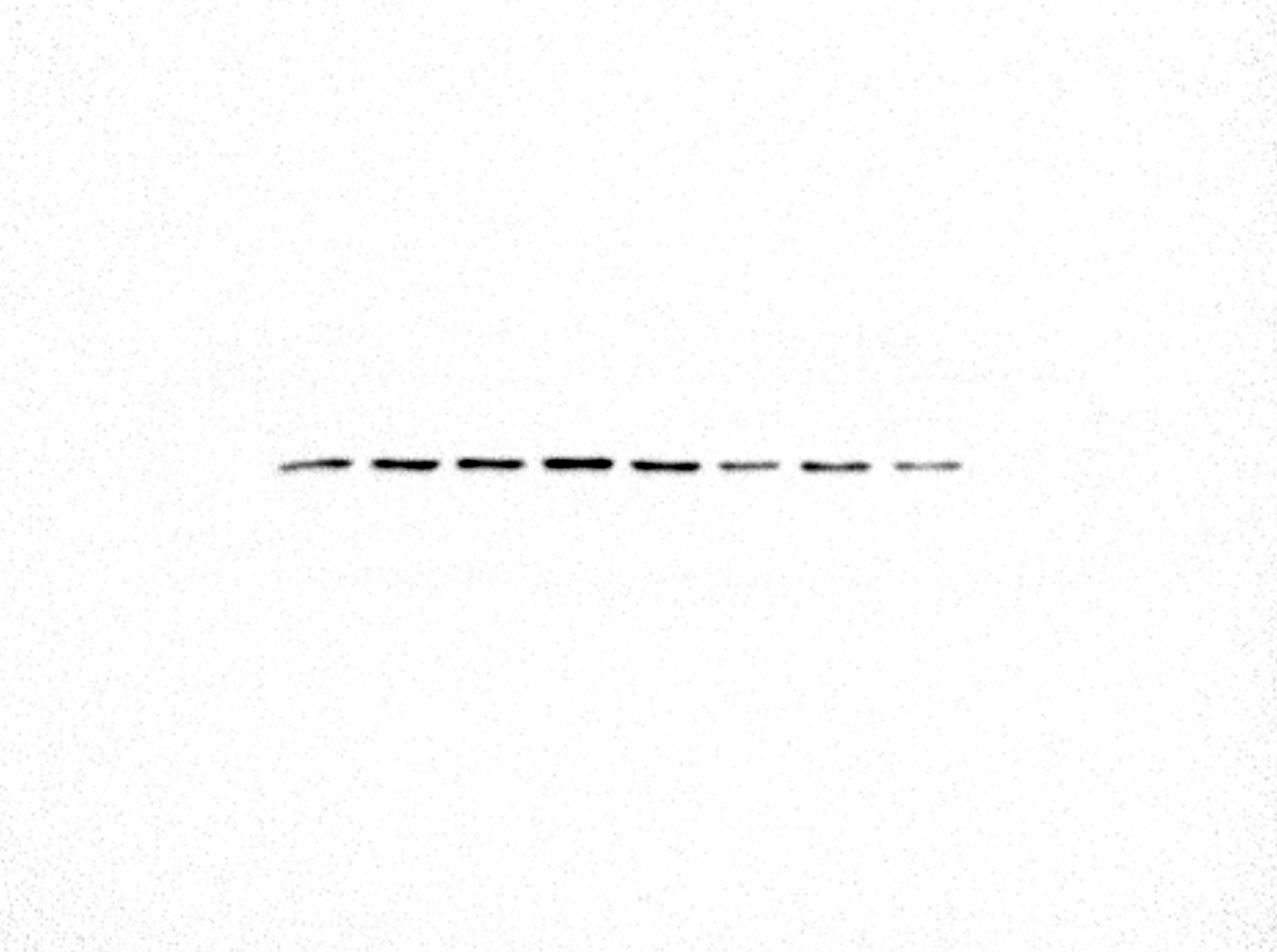

Supplement: Supplementary file 1 [file presentation1.zip › original image files/Figure 4B p-P38.tif]

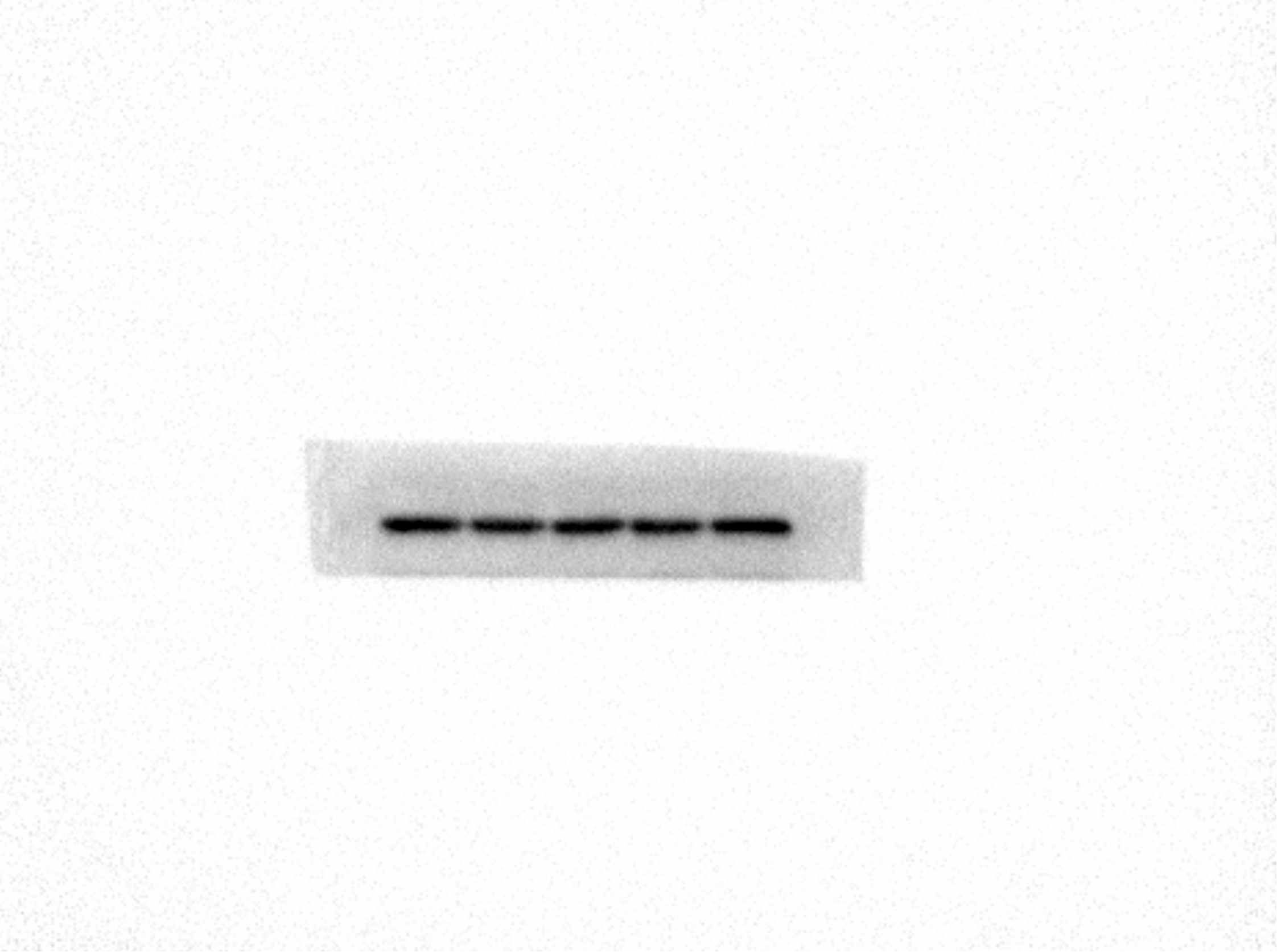

Supplement: Supplementary file 1 [file presentation1.zip › original image files/Figure 5C GAPDH.tif]

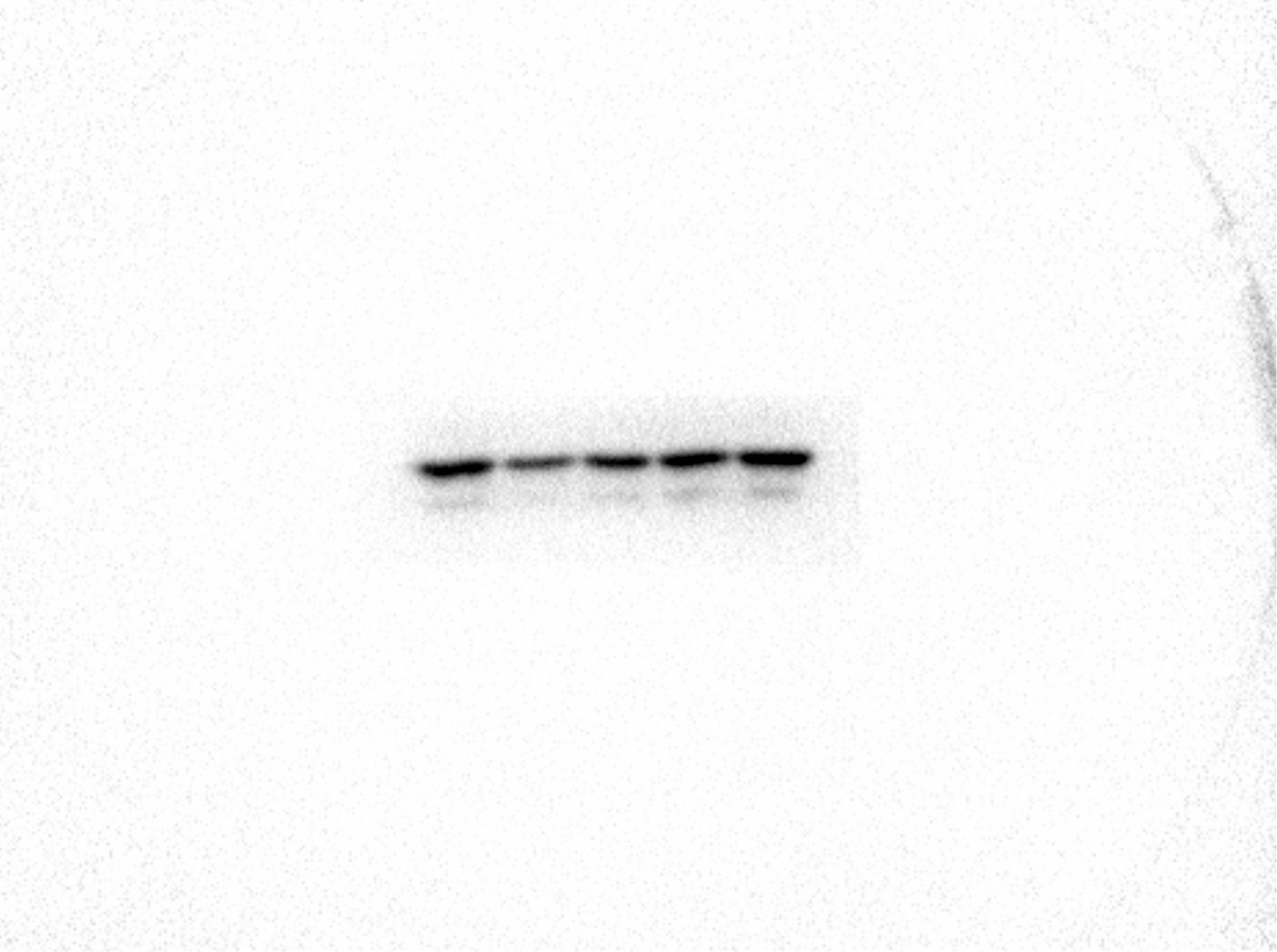

Supplement: Supplementary file 1 [file presentation1.zip › original image files/Figure 5C HO-1.tif]

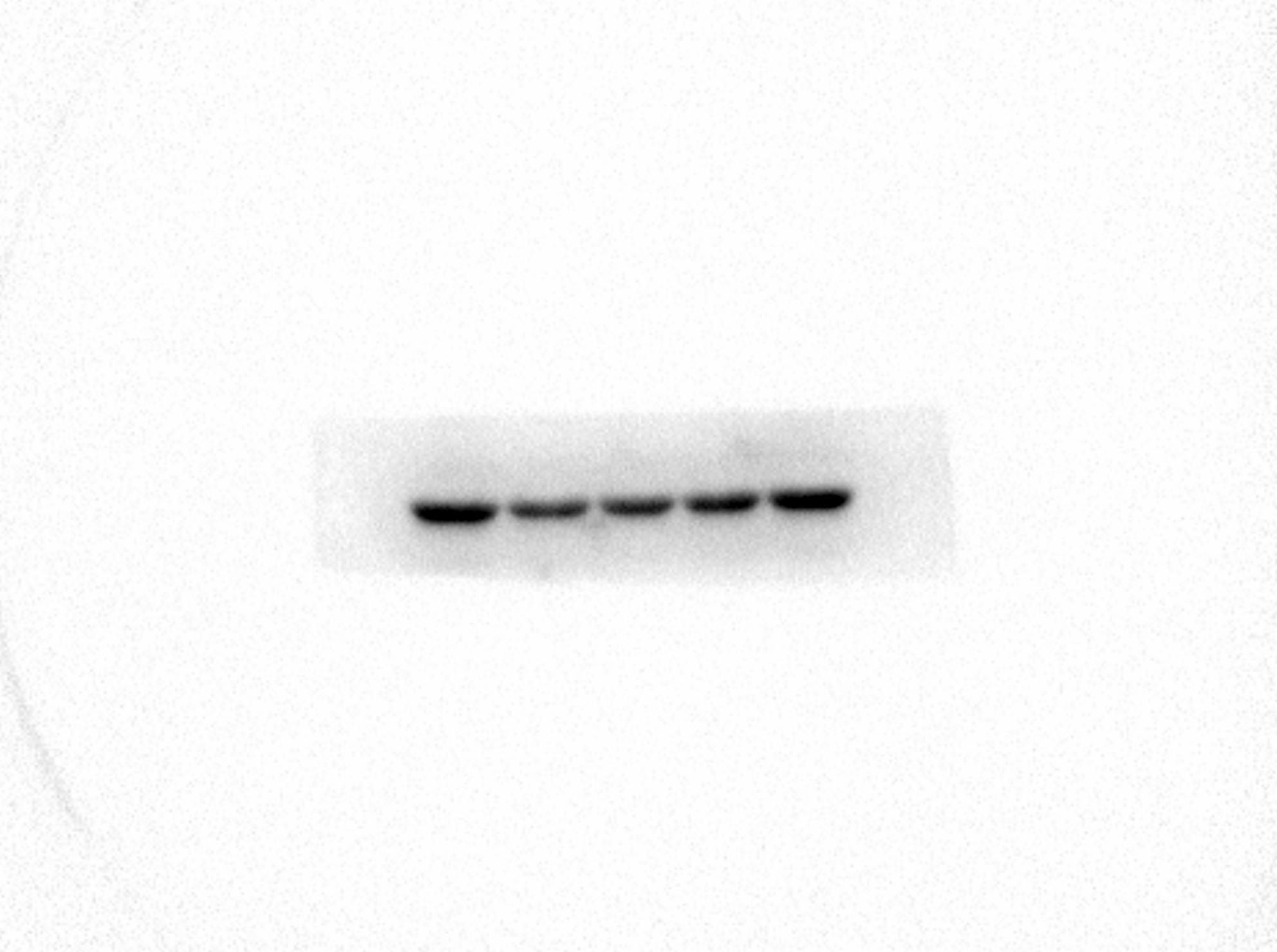

Supplement: Supplementary file 1 [file presentation1.zip › original image files/Figure 5C NQO-1.tif]

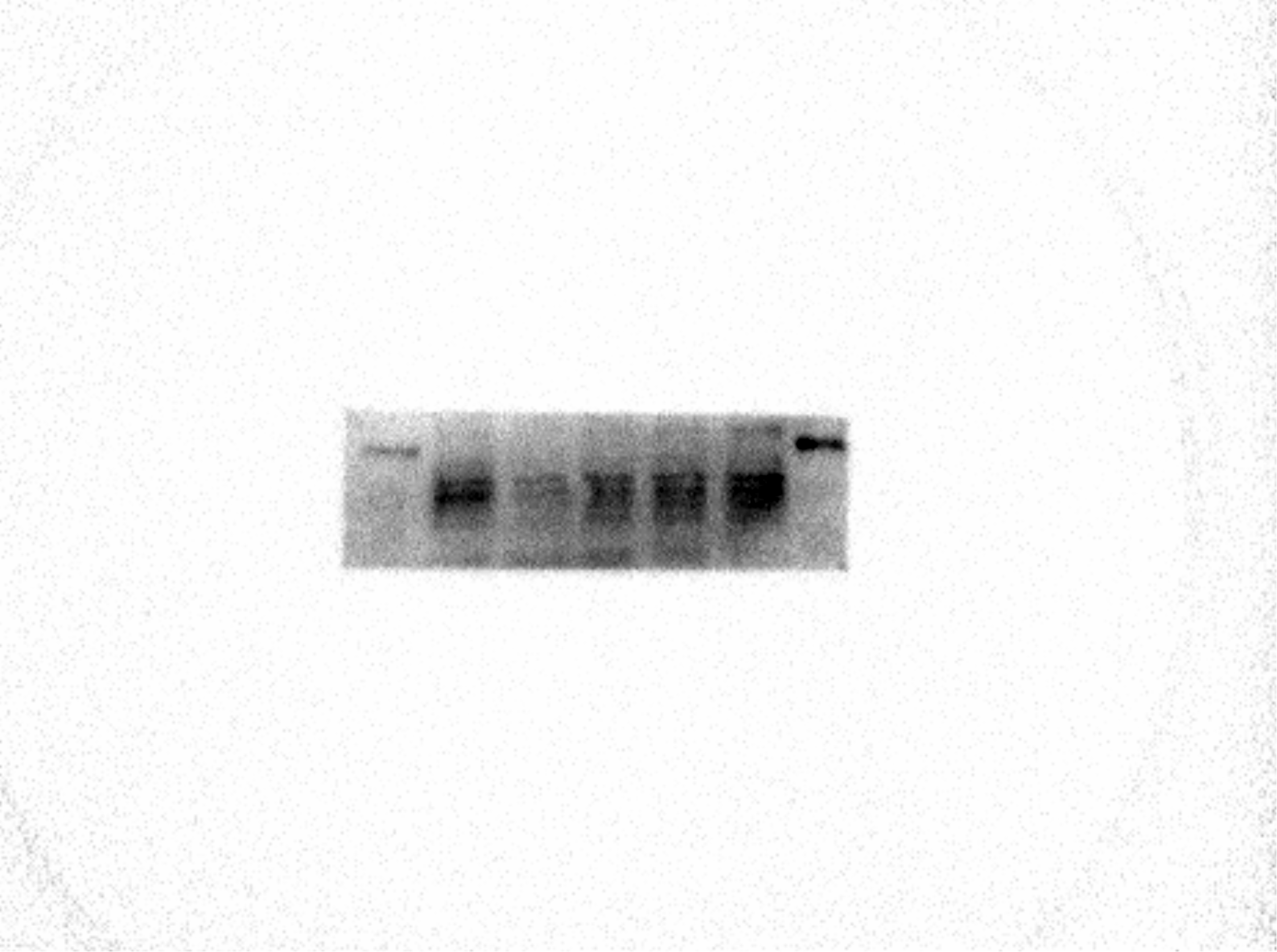

Supplement: Supplementary file 1 [file presentation1.zip › original image files/Figure 5C Nrf2.tif]

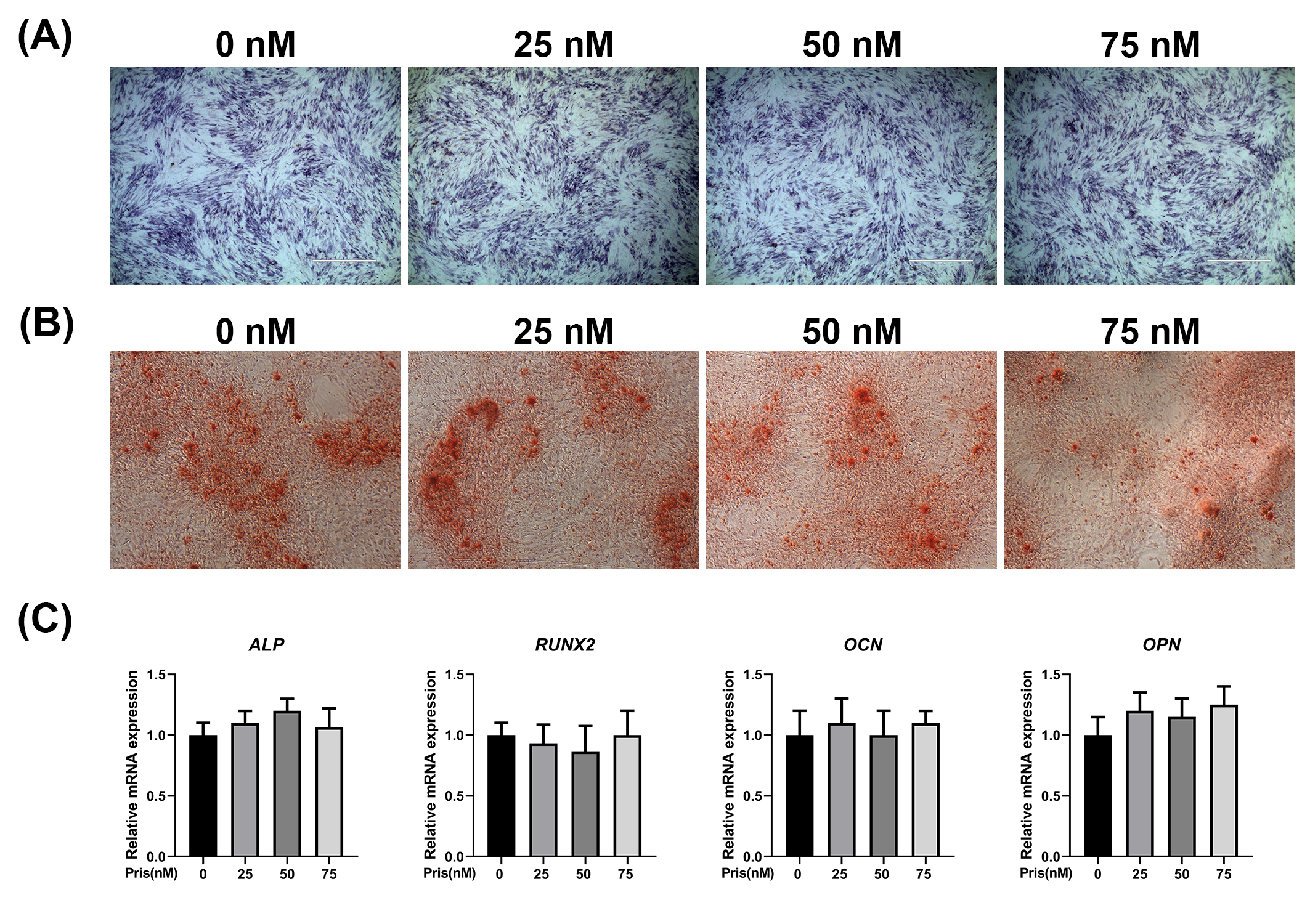

Supplement: Supplementary file 2 [file image1.tif]
